# The pathogen-encoded signalling receptor Tir exploits host-like intrinsic disorder for infection

**DOI:** 10.1038/s42003-024-05856-9

**Published:** 2024-02-13

**Authors:** Marta F. M. Vieira, Guillem Hernandez, Qiyun Zhong, Miguel Arbesú, Tiago Veloso, Tiago Gomes, Maria L. Martins, Hugo Monteiro, Carlos Frazão, Gad Frankel, Andreas Zanzoni, Tiago N. Cordeiro

**Affiliations:** 1 Instituto de Tecnologia Química e Biológica António Xavier, Universidade Nova de Lisboa, Av. da República, Oeiras, Portugal; 2 Department of Life Sciences, Imperial College London, South Kensington Campus, London, UK; 3 Department of NMR-supported Structural Biology, Leibniz-Forschungsinstitut für Molekulare Pharmakologie, Berlin, Germany; 4 Aix-Marseille Université, Inserm, TAGC, UMR_S1090, Marseille, France; 5 Present Address: InstaDeep Ltd, 5 Merchant Square, London, UK

**Keywords:** Intrinsically disordered proteins, Solution-state NMR, SAXS, Pathogens

## Abstract

The translocated intimin receptor (Tir) is an essential type III secretion system (T3SS) effector of attaching and effacing pathogens contributing to the global foodborne disease burden. Tir acts as a cell-surface receptor in host cells, rewiring intracellular processes by targeting multiple host proteins. We investigated the molecular basis for Tir’s binding diversity in signalling, finding that Tir is a disordered protein with host-like binding motifs. Unexpectedly, also are several other T3SS effectors. By an integrative approach, we reveal that Tir dimerises via an antiparallel OB-fold within a highly disordered N-terminal cytosolic domain. Also, it has a long disordered C-terminal cytosolic domain partially structured at host-like motifs that bind lipids. Membrane affinity depends on lipid composition and phosphorylation, highlighting a previously unrecognised host interaction impacting Tir-induced actin polymerisation and cell death. Furthermore, multi-site tyrosine phosphorylation enables Tir to engage host SH2 domains in a multivalent fuzzy complex, consistent with Tir’s scaffolding role and binding promiscuity. Our findings provide insights into the intracellular Tir domains, highlighting the ability of T3SS effectors to exploit host-like protein disorder as a strategy for host evasion.

## Abstract (web summary)

Tir, a bacterial effector, exploits host-like disorder to rewire cellular processes via dimerization, lipid binding, and phosphorylation, revealing a versatile intracellular strategy for infection.

## Introduction

Intrinsically disordered proteins and regions (IDPs and IDRs, respectively) are widespread across all kingdoms of life^1^. They do not adopt well-defined structures but are dynamic conformational ensembles that display unique properties complementary to globular proteins^2,3^. IDPs/IDRs play essential roles in signalling pathways^4^, including cell cycle^5^, circadian circuits^6^, post-transcriptional regulation^7^, and protein degradation^8^. Given their ubiquitous relevance in cellular signalling, the onset of several human diseases, including cancer and neurodegeneration, are linked to dysfunctional disordered proteins^9^. A hallmark of IDPs/IDRs is the high occurrence of short linear motifs, also known as eukaryotic linear motifs (ELMs)^10^. ELMs are stretches of 3–10 contiguous residues mediating transient protein-protein interactions (e.g., post-translational modification sites, targeting signals)^11^. For instance, phosphorylation sites are often found in protein IDRs, modifying charge and hydrophobicity and modulating partner interactions^7^. The prevalence of such functional elements in IDRs provides versatility to cell interaction hubs by enabling flexibility and adaptability to multiple interaction interfaces^12,13^. Moreover, IDR sequence composition often has low complexity and charged residues favouring electrostatic interactions at lipid bilayer surfaces^14^, with lipid-binding proteins accounting for 15% of all disordered proteins^15^. As a result, IDRs are common in membrane signalling receptors, particularly in their intracellular domains^16^.

Disordered proteins comprise 30-50% of eukaryotes’ proteins^17,18^. In contrast, they are rare in prokaryotes. However, increasing experimental evidence in the literature shows several effector proteins secreted by pathogenic bacteria containing functionally relevant IDRs^19^. An example is the *Helicobacter pylori* type IV secretion system effector CagA, which recruits host proteins to potentiate oncogenic signalling via a long IDR^20^. Another illustrative example is the effector CyaA of *Bordetella pertussis*, the causative agent of whooping cough that interacts with host calmodulin via a disordered stretch of 75 amino acid residues^21^. Other less-ordered effectors are also secreted by Enteropathogenic and Enterohemorrhagic *Escherichia coli* strains (EPEC and EHEC, respectively), such as EspF(U)/TccP^22^ and EspB^23^.

EPEC is a leading cause of child diarrhoea worldwide^24^, and EHEC causes hemolytic-uremic syndrome (HUS), defined by acute kidney failure, low platelet count, and destruction of red blood cells^25^. These bacteria attach to the gut mucosa, causing attaching and effacing (A/E) lesions, characterised by the formation of actin-rich pedestal-like structures and the brush border microvilli effacement. To establish a replicative niche on host cells, EPEC and EHEC deliver an array of T3SS effectors^26^. Here, we aimed to identify disordered effectors expressed by A/E pathogens. To this end, we ran a structural disorder analysis on the collection of effectors and searched for ELMs evidence. Among the disordered effectors, we found the prominent translocated intimin receptor (Tir) with a relatively high density of host-like ELMs, particularly in its intracellular domains. Tir is the first effector secreted during infection^27^. Once inside the host, it migrates to the plasma membrane to act as a receptor for the outer membrane bacterial adhesin intimin^28^, anchoring A/E pathogens to the plasma membrane of the host cells. Moreover, Tir promotes actin polymerisation^29,30^, suppresses autophagy^31^, and immune response^32^ to allow bacterial survival on the host cell surface. It also triggers pyroptotic cell death in macrophages^33^ and intestinal epithelial cells^34^. As a double-pass membrane receptor, Tir adopts a hairpin topology with the external intimin‐binding domain (IBD)^35^ connecting two transmembrane domains and both N- and C-terminus in the host cytoplasm (N-Tir and C-Tir, respectively). Those intracellular regions enable Tir to interact with at least 25 host targets^36^, such as host tyrosine phosphatases SHP-1/2 to suppress pro-inflammatory cytokines signalling^32,37^ and TAK1-mediated immune response^38^.

How Tir hijacks intracellular signalling by interacting with multiple host proteins to assist infection is poorly understood. To gain insights into Tir’s detailed biophysics and biological function, we devised a structural biophysical study to assess its intrinsic disorder and interactions using Nuclear Magnetic Resonance (NMR) and Small-angle X-ray scattering (SAXS) combined with AlphaFold-2^39^. We focus on stoichiometry, accessibility to multi-site phosphorylation, host protein- and membrane-binding of Tir intracellular regions. We found that N-Tir is a flexible dimer with a hybrid architecture of ordered and disordered parts brimmed with host-like ELMs. Moreover, we show that C-Tir is an IDR able to form phosphorylation-mediated fuzzy complexes with SH2 host domains that might facilitate binding promiscuity. C-Tir has non-random structures around host-like phosphorylation sites with lipid-binding ability pre-phosphorylation, suggesting a regulation based on the interplay between membrane association and phosphorylation. We uncover that Tir-induced actin polymerization and cell death depend on lipid-binding sites within disordered C-Tir.

Our findings highlight a molecular mechanism through which Tir can interact with host components by mimicking disordered host proteins, suggesting that pathogenic bacteria may share this strategy to assist infection.

## Results

### Structural disorder and short linear motifs are common features of A/E pathogen effectors

**Fig. 1:**
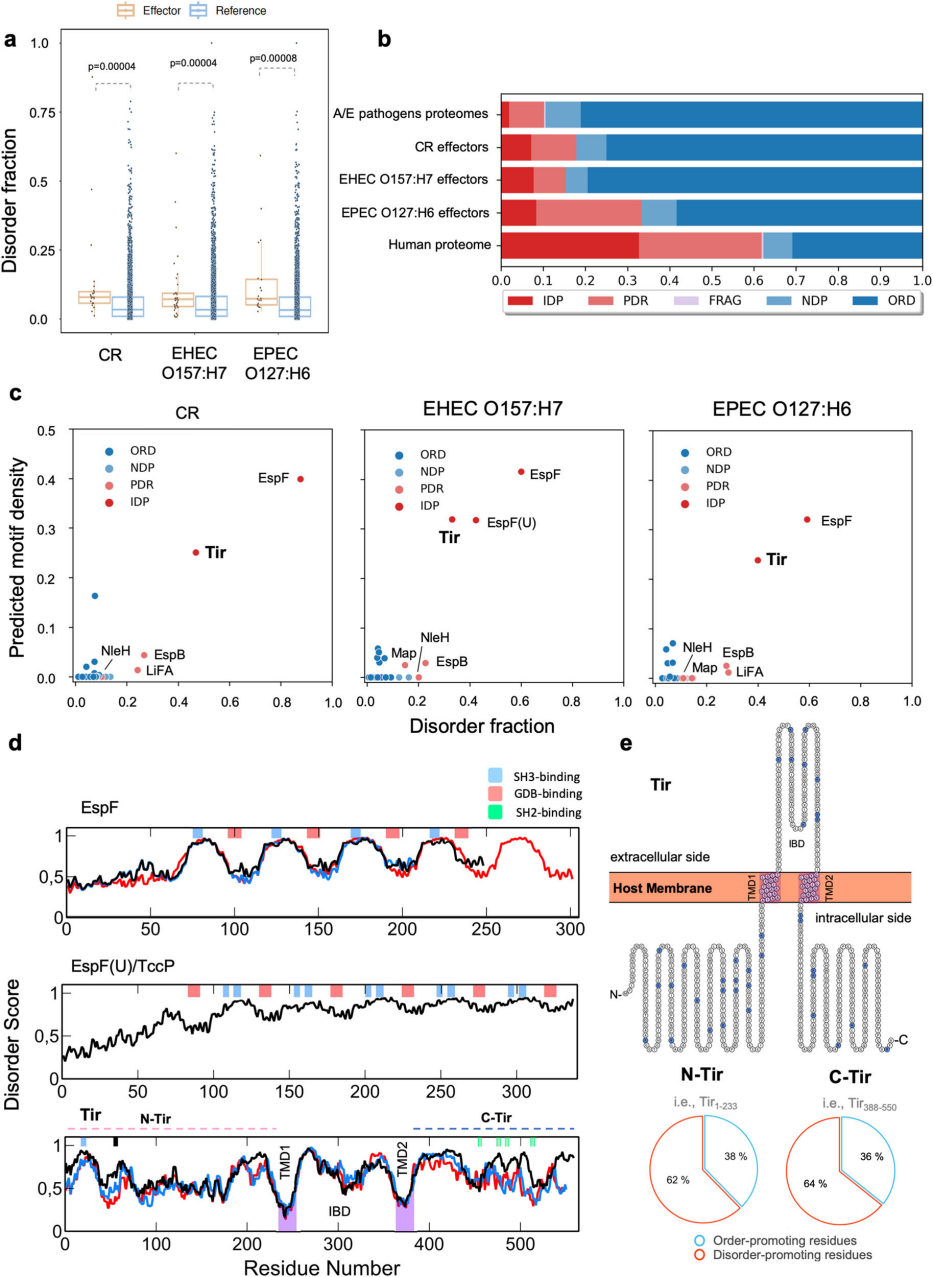
Predicted structural disorder in A/E pathogens. **a** Distribution of disorder fraction displayed as box plots for effectors (orange) and full-proteomes (blue) of three representative A/E pathogens. Statistical analysis with Mann-Whitney U-test. **b** Accumulated fractions of the structural categories in terms of structural disorder. IDP: Intrinsically disordered proteins; PDR: Proteins with intrinsically disordered regions; FRAG: Proteins with fragmented disorder; NDR: Not disordered proteins; ORD: Ordered Proteins. See definitions in Supplementary Table 2. **c** Fraction of ELMs vs. disorder fraction in A/E effectors. Tir, EspF, and EspF(U) display a high motif and disorder content. IDP and PDR effectors are labelled. See all data in Supplementary Table 3. **d** Disorder propensity of EspF (top), Espf(U)/Tccp (middle), and Tir (bottom) from EHEC O157:H7 (black line), EPEC O127:H6 (blue line), and CR strain ICC168 (red line). Tir’s disorder propensity is conserved among A/E pathogens. Experimental verified GBD-, SH3- and SH2-binding motifs are indicated in light red, blue, and green bars, respectively. The 14-3-3 motif within N-Tir first residues is indicated by a black bar. The transmembrane domains (TMDs) of Tir are in purple. **e** EPEC O127:H6 Tir in hairpin topology as determined using TopGraph^124^, with the IBD flanked by the two TMDs and cytosolic termini with high content in disorder-promoting residues (T, A, G, R, D, H, Q, K, S, E, P).

EPEC, EHEC and the mouse-specific pathogen *Citrobacter rodentium* (CR) are A/E pathogens. To quantify the prevalence of disorder propensity in these pathogens, we estimated the disorder content of their T3SS effectors using DISOPRED3^40^ (Fig. 1) and IUPred 1.0^41^ (Supplementary Figure 1). We observed that effector proteins have a higher disorder propensity than their proteome counterparts in EHEC O157:H7, the closely related EPEC O127:H6, and CR strain ICC168 (Fig. 1a, Supplementary Table 1). Subsequently, we classified the effectors into five structural categories based on disorder content (Supplementary Table 2**)**. Our analysis shows that A/E pathogens have a structurally diverse repertoire of effectors, ranging from fully unstructured to ordered proteins (Fig. 1b, Supplementary Table 3). While most proteins are folded in all prokaryotic collections (Supplementary Figure 2), IDP effectors are predicted to be two to four-fold more frequent than the whole bacterial proteome (1.9% vs. 7.7% on average). Partially Disordered Proteins (PDR) with long disordered regions occur in EPEC O127:H6 effectors at a similar frequency as the human proteome. For instance, we classified as PDR the EspB effector, involved in cytoskeleton rearrangement and previously identified as an inherently less-ordered effector^23^. An additional example of PDR is the NleH effector, a protein kinase with a disordered N-terminal domain that binds the ribosomal protein 3 (RPS3) to manipulate the NF-κB pathway^42^. In the order-to-disorder continuum, the effectors EspF and EspF(U)/TccP are IDPs (Fig. 1c). Both proteins contain multiple consecutive repeats of linear motif pairs critical to trigger actin assembly, with EspF having three to five repetitions depending on the strain^43^ and EspF(U)/TccP, only secreted by EHEC, five-and-a-half repeats^44^ (Fig. 1d). Our analysis further highlighted Tir as a disordered effector (Fig. 1c). Tir displays a high disorder propensity within its IBD and intracellular regions (Fig. 1d, e).

Pathogens can exploit host-like motifs as a strategy to interact with host components to subvert host cellular functions^45,46^. On this basis, we searched the A/E effectors for eukaryotic linear motifs (ELMs) and found that aside from EspF and EspF(U), other A/E effectors bear multiple putative ELM instances (Fig. 1c). Tir emerged again as having several predicted ELMs in its intracellular regions, including experimentally verified SH3- and SH2-domain binding motifs (Fig. 1d)^47^. Structural disorder and high ELM density support Tir’s ability to interact with several host proteins^36^. There is a correlation between disorder content and ELM density, being Tir, EspF(U), and EspF clustered with the highest disorder fraction and overall motif density (Fig. 1c), similar to eukaryotic IDPs^11,48^. This correlation highlights ELMs within disordered segments of bacterial effectors as a potential strategy to disrupt host networks.

### Tir’s N-terminal tail is a dimer with order-disorder duality

**Fig. 2:**
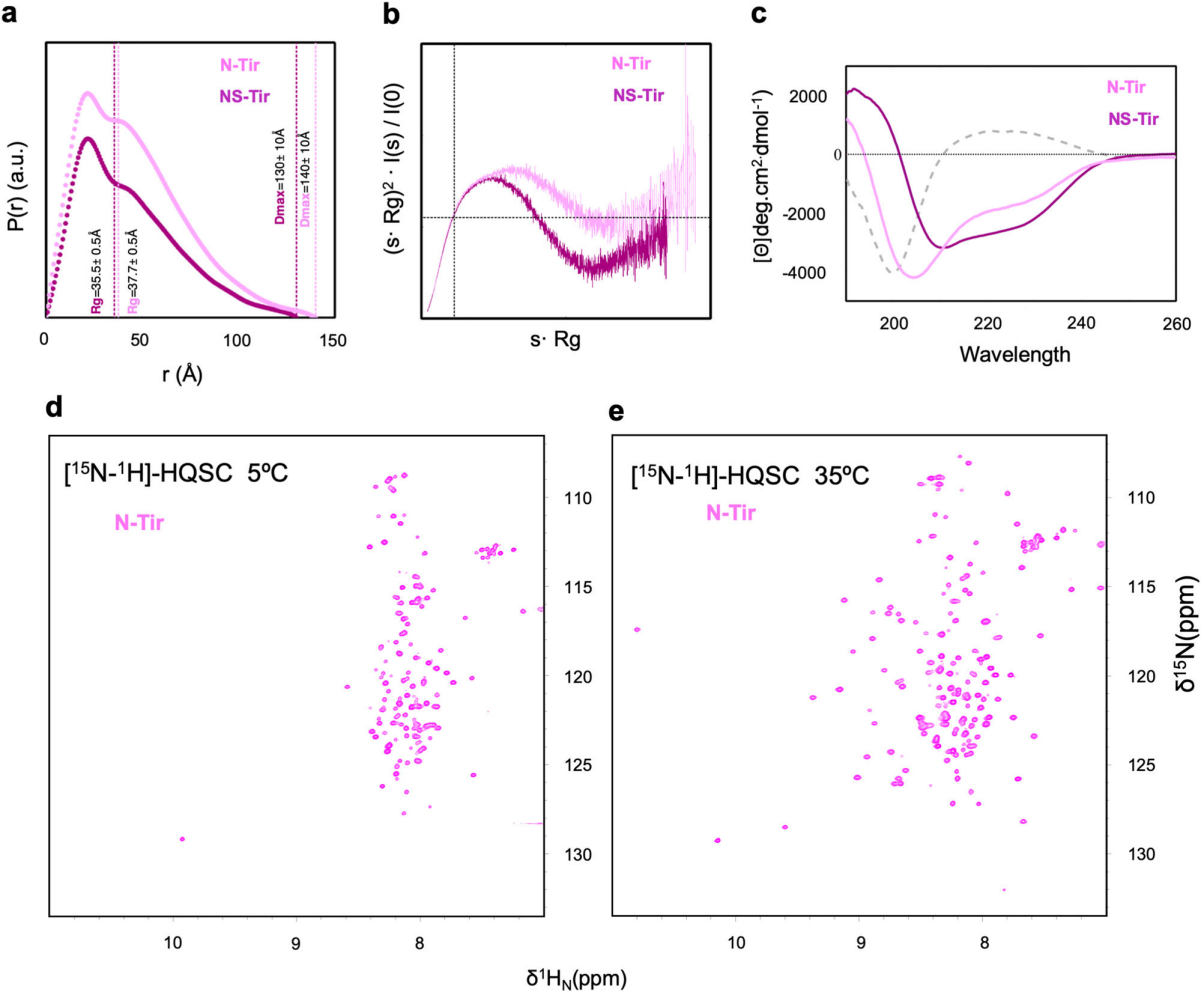
N-Tir is a partially disordered dimer. **a** Normalised pairwise distance distribution, *P(r)*, computed from experimental SAXS curves of N-Tir (pink) and NS-Tir (purple) consistent with an elongated dimer. Dashed lines indicate the derived *Rg* and *Dmax* values. **b** Kratky plots of N-Tir (pink) and NS-Tir (dark-magenta) highlight a high conformationally flexibility for N-Tir. **c** Far-CD spectra of N-Tir (pink) and NS-Tir (dark-magenta) and their difference (grey dashed line). **d** [^15^N-^1^H^N^]-HSQC spectra of N-Tir at 5 °C and **e** 35 °C reveal two dynamically different regions within the protein.

Tir shares substantial sequence conservation (Supplementary Fig. 3) and a high frequency of disorder-promoting residues^49^ at the N- and C-terminal regions (N-Tir and C-Tir, respectively), both localised in the host cytosol (Fig. 1e). We assessed the structural disorder propensity of EPEC O127:H6 N-Tir and C-Tir by multiple biophysical methods, including small-angle X-ray scattering (SAXS), circular dichroism (CD), and nuclear magnetic resonance (NMR). For this integrative study, we used the protein constructs encompassing the residues 1-233 and 388-550 from the N-terminal and C-terminal cytosolic regions of EPEC O127:H6 Tir, respectively (Fig. 1e, Supplementary Fig. 4, Supplementary Table 4). Based on size-exclusion chromatography (SEC)-SAXS data (Supplementary Fig. 5), the N-Tir is a non-globular protein with a radius of gyration (*Rg*) of 37.72 ± 0.50 Å and a maximum distance (*Dmax*) of 140.0 ± 10.0 Å (Fig. 2a, Supplementary Table 5), and a molecular weight compatible with a 52 kDa dimer, in line with the observed gel filtration retention time (Supplementary Figs. 4c and 5b). The respective *P(r)* curve is highly asymmetric and bimodal, with a peak at 21.9 Å and a prominent shoulder at 35.9 Å, suggesting that the dimer is elongated with spatially separated lobes. Its SAXS-derived Kratky plot is also not bell-shaped as expected for a non-globular and flexible arrangement (Fig. 2b). The corresponding SAXS-driven ab initio reconstruction highlights an elongated S-shaped core for the N-Tir dimer (Supplementary Fig. 6). This extended dimer is largely disordered but contains stable secondary structural elements. Its far-UV CD profile reveals a partially folded protein, marked by a negative maximum at 205 nm due to high disorder content. The positive signal at 190 nm and the negative shoulder at 220 nm reflect ordered elements (Fig. 2c). So, SAXS and CD data indicated that N-Tir is a partially folded dimer.

We have collected NMR data at different temperatures to probe the order-disorder interplay within N-Tir at high resolution. The 52 kDa dimer size is beyond the practical range amenable to traditional NMR spectroscopy in solution. The NMR signals of larger molecules relax faster, leading to line broadening, low spectral sensitivity, and eventually loss of NMR signals, specially at low temperatures. Nevertheless, we found that the 52 kDa dimer has a [^15^N-^1^H^N^]-HSQC NMR spectrum at 5 °C characterised by intense signals and low chemical shift dispersion akin to an IDP (Fig. 2d). We assigned the resonances at 5 °C mostly to the first 80 and last 35 residues of the N-Tir, meaning that those regions are flexible in solution. The missing resonances of the central residues became visible with increasing temperature due to faster tumbling (Supplementary Fig. 7a). At 35 °C, they adopted an NMR fingerprint of a folded-like protein (Fig. 2e*)*, reflecting a more well-structured region. With NMR, we could establish that N-Tir’s central sequence has the broad NMR fingerprint of a well-folded protein flanked by residues with poorly dispersed ^1^H^N^ backbone resonances pointing to disorder. A closer look at the structural disorder predictions computed for N-Tir also suggests an order-disorder duality, with flanking residues more flexible than those in the central region (Supplementary Fig. 7b). Such disordered segments have linear motifs experimentally identified to interact with host proteins **(**Fig. 1d)^47,50^. The partial deletion of these disordered regions reduced the random-coil content reported by CD data, highlighting a more structured central part of N-Tir (N-Tir_61-200_, hereafter NS-Tir, standing for N-Terminal structured region of Tir). The difference between CD spectra resulted in a pure random-coil CD signature, reinforcing that the flanking regions are disordered. NS-Tir’s CD profile displays positive values below 200 nm and two negative bands at 210 and 222 nm, commonly associated with structured secondary conformations (Fig. 2c). We have employed SAXS to probe further the overall structure of NS-Tir. Our synchrotron SEC − SAXS data confirmed that NS-Tir is still a stable dimer in solution. It has a slightly less broadened Kratky profile that reflects the absence of the disordered flanking regions (Fig. 2c) but is compatible with an elongated dimer retaining a similar overall conformation as supported by NMR. We acquired [^15^N-^1^H^N^]-HSQC data for NS-Tir and superimposed them to N-Tir spectra (Supplementary Figure 7c). Over 75% of the visible resonances overlapped between the two constructs, with a low dispersion for the ^1^H dimension at 5 °C and a broader [^15^N-^1^H^N^]-fingerprint at 35 °C, indicating that NS-Tir maintained a similar folded isolated and in the full-length N-terminal region context. The nearly reversible thermal denaturation of NS-Tir monitored by far-UV CD shows the dimer unfolding in two discrete steps, capturing both folding and dimerization events. It reveals a stable dimer that unfolds and dissociates by increasing temperature^51^ (Supplementary Figure 8). Our data firmly supports that N-Tir is partially disordered with a stable dimer core.

### N-Tir encodes a central antiparallel OB-like fold dimer

**Fig. 3:**
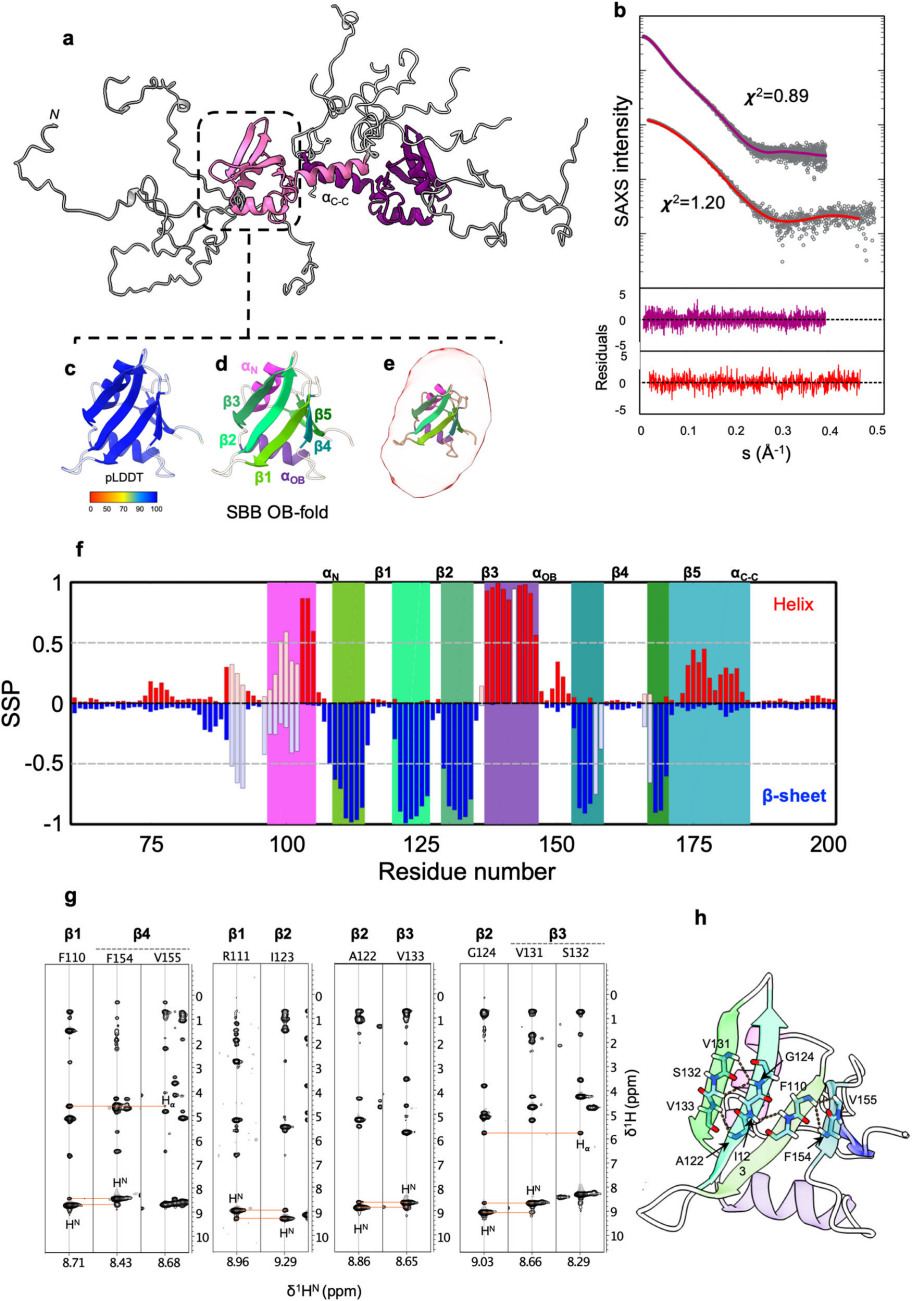
NS-Tir encodes a small OB-fold domain flanked by IDRs. **a** Structural ensemble of the NS-Tir antiparallel dimer stabilised by coiled-coil interaction flanked by disordered segments. N-Tir central region encodes for a small β-barrel (SBB) OB-fold domain. **b** SAXS intensity from NS-Tir (top) and SBB-Tir (bottom), *I(s)*, represented in logarithmic scale as a function of the momentum of transfer, s = (4πsin θ)/λ, where 2π is the scattering angle. The solid lines correspond to the scattering profile calculated from the EOM-selected NS-Tir ensemble (purple line, χ2 = 0.89) and the ab initio model of SBB-Tir (red, *χ*^2^ = 1.20), best fitting the experimental data. Point-by-point residuals of fittings and the absolute intensities are shown at the bottom. **c** Ribbon representation of the best AF2-based model for the SBB domain with colouring according to the predicted local difference test (pLDDT) metric, from high in blue (>90) to yellow (70), orange (50) and low in red (0). **d** The structural elements (SSEs) are coloured into N-Tir’s SBB model, highlighting an OB-fold topology (N-term → C-term: β1-β2-β3–α_OB_-β4–β5). **e** Superposition of the ab initio envelope (transparent red) and the respective AF2 model, with SSEs coloured. **f** Secondary structure propensity for β-strands (blue) and helices (garnet) calculated from protein backbone chemical shifts using N-Talos^57^. Highlighted in colour are the SSEs of the AF2-models. Transparency has been added to the 16 residues which could not be assigned. **g** Strips from a ^1^H,^15^N]-NOESY-HSQC spectrum showing cross-strand NOEs (orange dashed lines) between amide and backbone protons compatible with antiparallel beta-sheets. **h** Cross-strand NOEs (orange dashed lines) are displayed on the SBB domain structure.

To gain insight into the structure of N-Tir’s dimer core, we turned to AlphaFold-2 (AF-2)^52^ and Flexible Meccano (FM)^53^ to create a structural ensemble model of the NS-Tir dimer (Fig. 3a) consistent with SAXS data (Fig. 3b). AF-2 is an AI-based method that predicts a protein’s 3D structure from its amino acid sequence. It regularly achieves high accuracy competitive with experimental NMR structures^54,55^. We obtained, with high confidence (pLDDT 93.02 ± 8.92), that the central residues 97–171 form a small β-barrel (SBB) domain^56^ (Fig. 3c). The predicted features show five strand fragments (β1, residues 109 to 114; β2, residues 120 to 126; β3, residues 129 to 134; β4, residues 153 to 156; β5, residues 168 to 170) which form the SBB, that together with an intercalated helix (αOB, residues 137 to 146) and the N-terminal helix (αN, residues 97 to 105), adopt an oligonucleotide/oligosaccharide-binding (OB)-like fold topology (Fig. 3d) well-encapsulated by its SAXS ab initio reconstruction (Fig. 3e). Moreover, chemical shift analysis^57^ confirms the secondary structure topology (N-term → C-term: β1-β2-β3–α_OB_-β4–β5), with the secondary structural elements matching those obtained using the experimental chemical shifts of NS-Tir (Fig. 3f). To reinforce and clarify this finding, we show the expected signature of intramolecular cross-strand NOEs for the SBB domain. In Fig. 3g, the NOE spectrum shows a set of unambiguous long-range NOEs between β1 and β4, β1 and β2, and β2 and β3, which, in combination with their secondary chemical shifts, point to the presence of an antiparallel β-barrel within this region (Fig. 3h). The predicted OB-fold domain is followed by an α-helix (172-187, α_C-C_) seemingly interacting with the corresponding α-helix from another molecule to assemble an antiparallel coiled-coil homo-dimer (Fig. 3a**)**. To complete the model, we modelled the flanking regions as flexible ensembles with FM (Fig. 3a), obtaining structures that reproduce the experimental scattering profile (Fig. 3b), thus reinforcing the overall architecture of the NS-Tir as an ordered-disordered dimer. We disrupt the dimer by adding a stop codon in the middle of the α_C-C_ (at position 181), producing a stable monomer as seen experimentally by SEC-SAXS, thus implying the involvement of α_C-C_ in dimerization. This construct, encoding for residues 76-180 of Tir, bears the predicted SBB without the α_C-C_ (N-Tir_76-180_, hereafter SBB-Tir). The synchrotron SEC − SAXS data for SBB-Tir is compatible with a globular protein with an *Rg* of 17.8 ± 0.8 Å and a *Dmax* of 70 ± 10 Å, and the molecular weight of a monomer (Supplementary Table 4), in line with the observed gel filtration retention volume. Our results suggest a model of Tir spanning the host plasma membrane with partially disordered N-terminal intracellular domains assembled in an antiparallel dimer bearing an OB-like domain and dangling ELMs.

### Tir bears an intrinsically disordered C-terminal tail

**Fig. 4:**
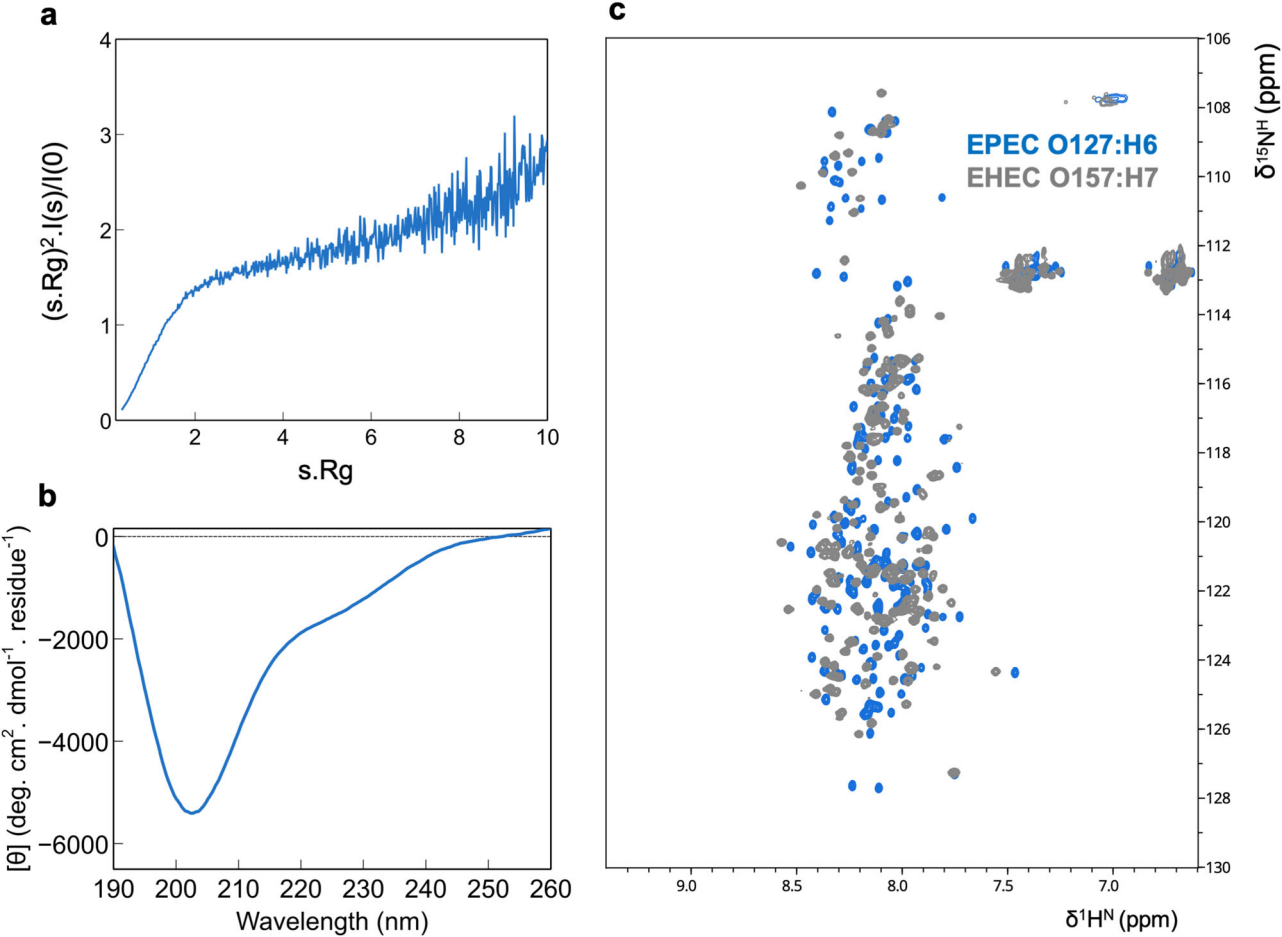
The Tir C-terminus is intrinsically disordered. **a** Kratky representation of the SAXS profile measured for C-Tir indicates a lack of compactness. **b** Far-UV circular dichroism of C-Tir reveals the absence of high-populated structural secondary elements. **c** C-Tir EPEC O127:H6 (light blue) and C-Tir EHEC O157:H7 (grey) [^15^N-^1^H^N^]-HSQC spectra reveal the narrow ^1^H chemical shift dispersion characteristic of IDPs with ^1^H amide backbone resonances clustering between 7.7 and 8.5 ppm. All together provide definite proof of protein disorder.

We also evaluated the structural disorder propensity of C-Tir. However, contrary to N-Tir, this region lacks any fixed-structured elements. Analytical SEC in combination with SAXS reveals a monomeric state for C-Tir in solution with an *Rg* of 38.8 ± 0.2 Å and a *Dmax* of 128.0 ± 5.0 Å (Supplementary Figure 6, Supplementary Table 5). The *Rg*-values of IDPs approximately followed a power law of *Rg* = 2.54*N*^0.522^ ^58,59^ as a function of sequence length (*N*). Using this relationship, the predicted *Rg*-value is 37.4 Å for the 173-residue C-Tir fused with a StrepTag (8 extra residues). The similarity between the experimental and predicted values suggests that C-Tir adopts IDP-like structures in solution. The SAXS-derived Kratky plot of C-Tir also shows characteristics of disordered or unfolded proteins, monotonically increasing without a well-defined maximum (Fig. 4a). This feature is absent in globular proteins and implies conformational heterogeneity and flexibility for C-Tir. Likewise, the asymmetric pair distance distribution function, *P(r)*, obtained from the scattering data, is compatible with a highly flexible protein sampling pairwise distances far exceeding those expected for a globular protein of the same molecular weight^59^ (Supplementary Figure 6). The intrinsic disorder of C-Tir is also reflected in its CD and [^15^N-^1^H^N^]-HSQC NMR spectra. C-Tir’s CD profile has negative ellipticity at 200 nm and a shallow band in the 210–230 nm range (Fig. 4b), indicating a high content of random coil with minimal ordered structural elements^60^. This construct’s 2D-[^15^N-^1^H^N^]-HSQC NMR spectrum displays a typical IDP fingerprint with low chemical shift dispersion^61^ (Fig. 4c). We observed a similar spectrum for the equivalent region of Tir from EHEC (Fig. 4c), highlighting its structural disorder’s conservation and functional importance for A/E virulence. Overall, the different biophysical measurements are consistent with a highly dynamic protein, unambiguously showing that the C-Tir is an IDR that lacks a well-folded structure. Such inherent flexibility will potentially enable Tir for multiple binding while acting as a scaffold/hub of intracellular host proteins.

### C-Tir displays non-random structural preferences at phosphorylation sites

**Fig. 5:**
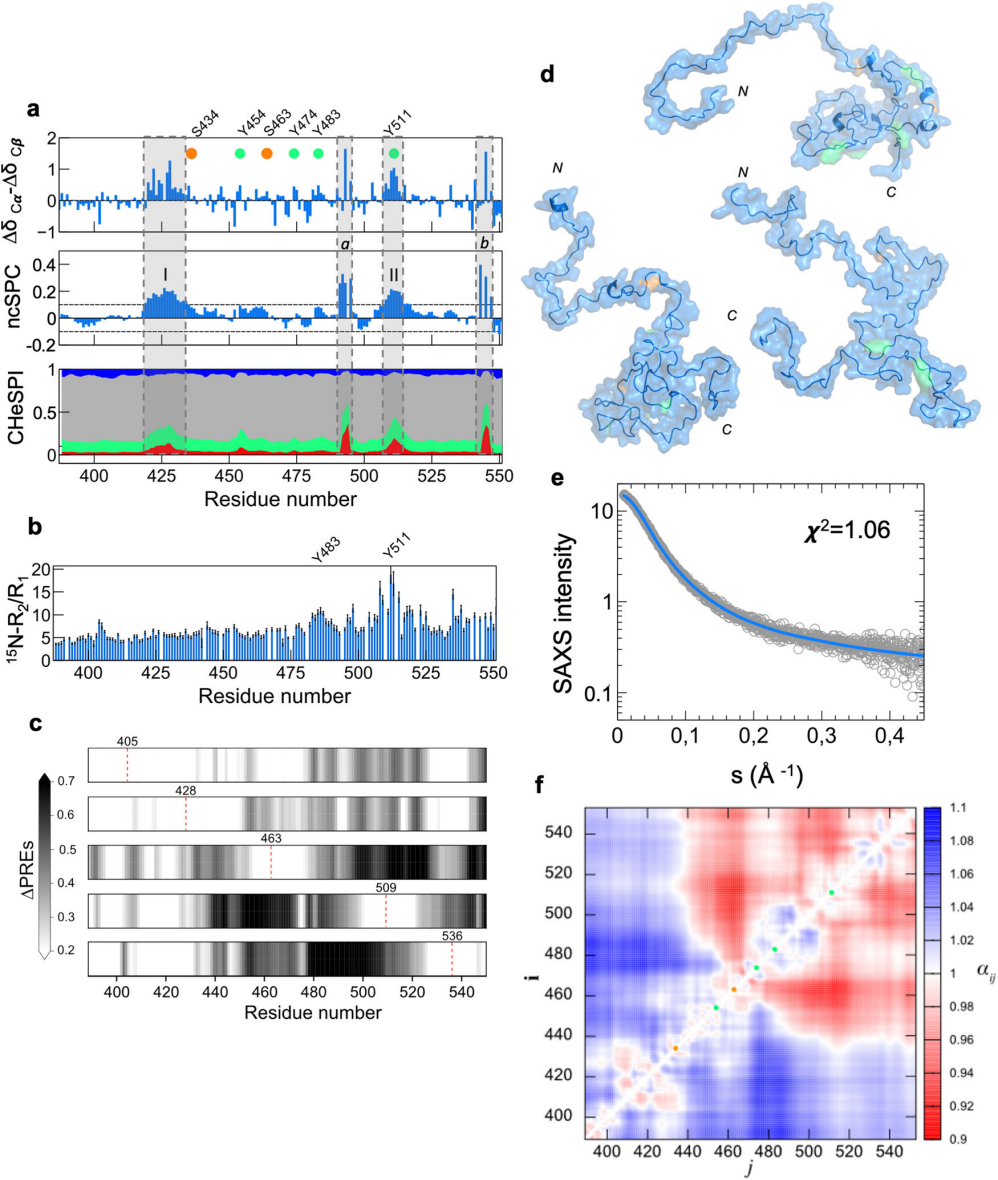
C-Tir is disordered with residual structure at sites of host interaction. **a** Secondary chemical shifts (∆δCα-ΔδCβ) (top), structural propensity plot (middle) for C-Tir where the dashed-lines depict the random-coil threshold and stacked bar plot of CheSPI populations (bottom) of extended (blue), helical (red), Turn (green), and non-folded (grey) local structures. ncSPC values above or below this threshold have ɑ-helix or β-sheet propensities, respectively. Positive values (grey regions) show an increased α-helix tendency at segments D420-Q435 (I) and I507-A515 (II), as well as F489-V495 (*a*) and T543-V547 (*b*). **b** ^15^N–R_2_/R_1_ analysis of C-Tir. **c** ΔPRE-values measured on V405C, S428C, S463C, S509C, and S536C C-Tir mutants as grey-scale heatmaps, from top to bottom, respectively. Color intensity reflects the relative deviations between the experimental PRE and those predicted from a random-coil model (ΔPRE = [I_para_/I_dia_]_*RC*_ - [I_para_/I_dia_]_*exp*_). Dashed red lines mark the position of MTSL tags. **d** Cartoon representations of 3 representative conformers of the C-Tir ensemble refined with PRE + SAXS data with serine- and tyrosine-phosphorylation sites colored orange and green. **e** Logarithmic-scale of scattering intensity, *I(s)*, as a function of the momentum transfer, *s*, measured for C-Tir (empty gray circles). The solid line is the averaged back-calculated curve derived from the PRE + SAXS-based ensemble of C-Tir (blue). **f** Ensemble-averaged contact map normalized to random-coil distances (α_*ij*_ = 〈*r*_*ij*_〉_*ens*_/〈*r*_*ij*_〉_*RC*_). Green and orange circles mark phosphorylation sites on C-Tir for tyrosine and serine residues, respectively. C-Tir is bipartite, with its C-terminal half being more compact.

We employed NMR to dissect with further detail the structural features of C-Tir. The chemical shift (CS) is the most readily observable in NMR and sensitive to even partial secondary structural elements present in IDPs^61^. To access this information, we assigned the backbone resonances of C-Tir nuclei using standard triple-resonance spectra at high-field and reverse labelling (Supplementary Figure 9). Reverse labelling of selected amino acids^62^ proved to be a cost-efficient approach to assign this disordered protein by reducing the spectral ambiguity and should be readily applied to other IDPs. Given the assignment, we calculated the CS deviations (∆δ) to random coil values of intrinsically disordered proteins^63^. Although C-Tir has properties of a random coil, these so-called NMR secondary shifts allowed us to identify two regions with non-random structural preferences. In IDPs, transient secondary structural elements are often crucial molecular recognition units, playing critical roles in binding^64,65^. In Fig. 5a, the consecutive positive ΔδCα–ΔδCβ values indicate a tendency for α-helical structure in two distal segments of C-Tir: (I) D420-Q435; II) I507-A515. Both stretches include residues phosphorylated by host kinases, S434^66^ and Y511^32^, respectively. Considering all the assigned backbone resonances in a neighbour-corrected sequence structural propensity calculator (nsSPC)-score^67^ made these local α-helical regions more evident. New stretches also emerged with a putative α-helical propensity, including a sequence contiguous to Y511 and downstream tyrosine 483 (Fig. 5a). We also employed the CheSPI (Chemical shift Secondary structure Population Inference) method^68^ to provide additional local structure and disorder assignment. CheSPI analysis (Fig. 5b) further confirmed that C-Tir is disordered with small fractions of folded structural elements (i.e., helix, turn) at the residues with higher nsSPC-score. ^15^N-relaxation measured on C-Tir also supports the existence of less-disordered segments near Y483 and Y511 with relatively elevated ^15^N-R_2_/R_1_. Interestingly, Y483 and Y511 are core residues of Tir’s host-like immunoreceptor tyrosine-based inhibitory motifs (ITIMs) that recruit tyrosine phosphatases SHP-1/2 in an ITIM phosphorylation-dependent manner to inhibit host innate immune responses^32,37^.

We further probed C-Tir’s non-random coil preferences using paramagnetic relaxation enhancement (PRE) NMR experiments. To this end, five residues scattered along C-Tir’s sequence were single-point mutated to cysteine to attach a thiol-reactive nitroxide radical probe (i.e., MTSL). The relaxation induced by this paramagnetic tag resulted in substantial peak broadening (Supplementary Figure 10). This effect only affects residues near the MTSL tag in ideal random coils. Yet, if long-range contacts are present within the disordered chain, residues far away in the sequence can be spatially close to the tag and experience the PRE effect. We calculated the deviations between the experimental PRE and those predicted from a random-coil model (i.e., ∆PRE) to detect non-random coil structural preferences. We measured ∆PRE-values for each single-cysteine mutant. Figure 5c shows, in a grayscale, the ∆PRE induced by MTSL at positions 405, 428, 463, 509, and 536, all consistent with long-range compact conformations in the disordered C-Tir. The paramagnetic probe in positions 405 and 428 showed noticeable long-range interactions (∆PRE > 0.2) with distant downstream residues. The MTSL at S463C caused substantial PRE (∆PRE > 0.6) in the residues 497–526, and when placed in position 509, it induced broadening in the region 438-479, including position 463. These complementary long-range interactions observed between residues around positions 463 and 509 are mutually self-consistent with compact conformations. Interestingly, both areas include tyrosine-based ELMs involved in intracellular signalling. To better describe and visualise the non-random preferences of C-Tir, we integrated the PRE data with SAXS data to determine the conformational ensemble of C-Tir (Fig. 5d). The established sub-ensembles agree with the experimental data (Fig. 5e, Supplementary Figure 10), revealing a bias for expanded and compact structures. The ensemble-averaged distances between the Cα atoms for the experimentally selected conformations (〈*r*_*ij*_〉_*ens*_), normalised by the respective random coil (RC) ensemble (〈*r*_*ij*_〉_RC_), show an N-terminal region more extended relative to the RC. At the same time, the C-terminal half is more compact (Fig. 5f), highlighting a bipartite nature. Compaction is clustered around the host-like ITIMs that interact with host proteins.

### C-Tir can interact with the host SHP-1 pre-phosphorylation

**Fig. 6:**
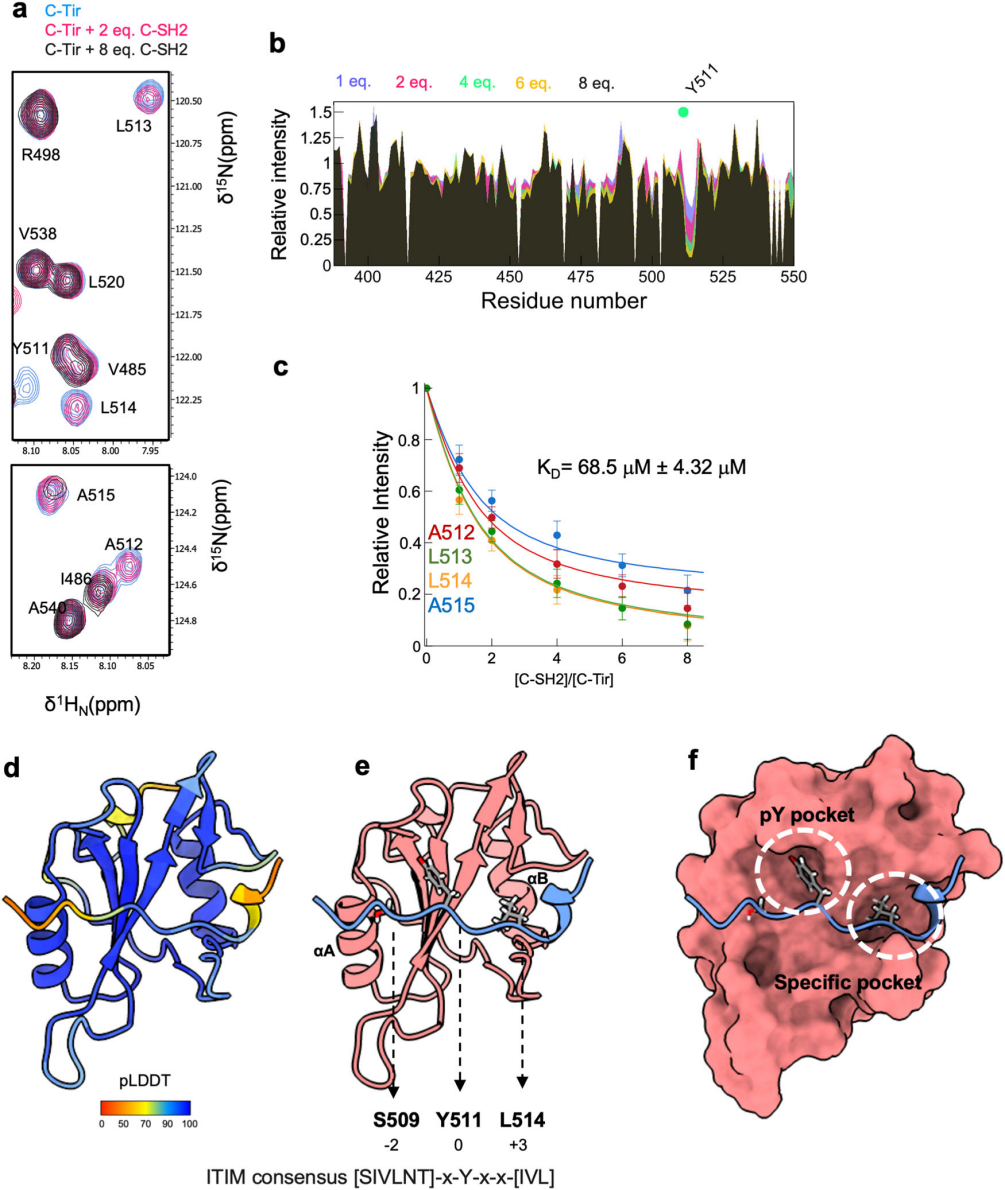
C-Tir can bind host C-SH2 domain of SHP-1 pre-phosphorylation. **a** Overlay of [^15^N-^1^H^N^]-HSQC spectra of C-Tir in the absence (blue) and the presence of C-SH2 at 2.0 (pink) and 8.0 (dark grey) equivalents. We show the NMR signal of residues A_512_LLA_515_ with an intensity drop due to C-SH2 binding. **b** Relative [^15^N-^1^H^N^]-peak intensities from the titration analysis. The green circle marks the tyrosine Y511 position. **c** Global fitting NMR quantification of the binding of C-SH2 to unphosphorylated C-Tir. The data shown are normalized intensities by the initial value. The error bars are the uncertainty from random noise in the ratio of two peak heights. The solid lines correspond to the fitting. **d** Ribbon representation of the best AF2-based model for the Y511 motif in complex with C-SH2 from SHP-1 with colouring according to the pLDDT metric. **e** Scheme of the binding interface between an SH2 domain (in pink) and the unphosphorylated Y511 peptide (in blue). Y511 and other interface residues are in stick format. The peptide is numbered by counting Y511 as 0. **f** Dashed white circles indicate the pY binding pocket and the specificity pocket.

The intracellular signalling associated with Tir ITIM-like sequences relies on the phosphorylation of the central tyrosine by Src family protein tyrosine kinases (PTKs), creating a binding site for SH2–containing proteins, which become activated upon recruitment. Nevertheless, unphosphorylated C-Tir can recognize host SH2-domains with low-intermediate affinity. Using the C-terminal SH2 domain (C-SH2) of phosphatase SHP-1, we identified, by NMR, that residues surrounding the unphosphorylated Y511 ITIM-like motif are involved in binding SH2 domains (Fig. 6a). Binding to C-Tir caused a selective loss of the backbone amide cross-peaks intensities, mainly from residues A_512_LLA_515_ (Fig. 6b). Visible NMR signals retained low dispersion, indicating that the protein remains mostly disordered and flexible in the complex. We estimated an apparent K_D_ of *ca*. 68.5 μM for this interaction (Fig. 6c). Thus, besides encoding for less-disordered structures, the sequence surrounding Y511 (i + 4) has a chemical signature for C-SH2 binding pre-phosphorylation. To gain insight into the complex interface, we harvest the capability of AF-2 to accurately model protein-peptide interactions^69^. In that sense, we employed Alphafold2-multimer^39^ using the sequence stretch of the unphosphorylated C-Tir mapped by NMR to bind C-SH2, obtaining a complex with high local and inter-chain accuracy (Fig. 6d, Supplementary Figure 11). The SH2 domain shows its typical central β-sheet flanked by two α-helices (αA, αB) (Fig. 6e). The Y511-motif binds perpendicularly to the β-sheet and docks into two abutting recognition sites formed by the β-sheet with each of the α-helices, similar to the complex of C-SH2 with the peptide NKG2A (PDB 2YU7). The Y511 points to the canonically defined “pTyr pocket”, and the SH2 recognizes the hydrophobic Leu residue in position +3 via the “specific pocket”^70^. Moreover, residue in position −2 interacts with αA, forming a “three-pronged plug” interaction altogether. This binding mode was observed for the SH2 domain of the SLAM-associated protein (SAP). Surprisingly, the SH2 SAP also recognizes unphosphorylated motifs due to the additional binding site, but with a lower affinity than the sequences containing pTyr residues^70,71^.

### Multi-phosphorylated C-Tir is a disordered multivalent cytosolic tail

To fully assess Tir ability to recruit SH2 domains, we reconstructed C-Tir’s Tyr-phosphorylated state and evaluated its binding to C-SH2. To this end, we incubated C-Tir with the Src family PTK Fyn, and quantitatively monitored its phosphorylation by NMR^72^. The sensitivity of chemical shifts to changes caused by phosphorylation allowed us to identify four phosphorylated tyrosine sites along the disordered C-Tir modified by Fyn (i.e., Y454, Y474, Y483, Y511). Phosphorylation caused small but noticeable changes in the chemical shifts of the tyrosines and adjacent residues. The [^15^N-^1^H^N^]-HSQC spectrum displayed low amide proton dispersion, a diagnostic that the C-Tir remains disordered upon multisite phosphorylation. Moreover, the secondary chemical shifts of this 4-fold phosphorylated state (pC-Tir) did not reveal substantial changes in local structure propensity due to phosphorylation (Supplementary Figure 12), as similarly reported for other disordered proteins^73,74^.

**Fig. 7:**
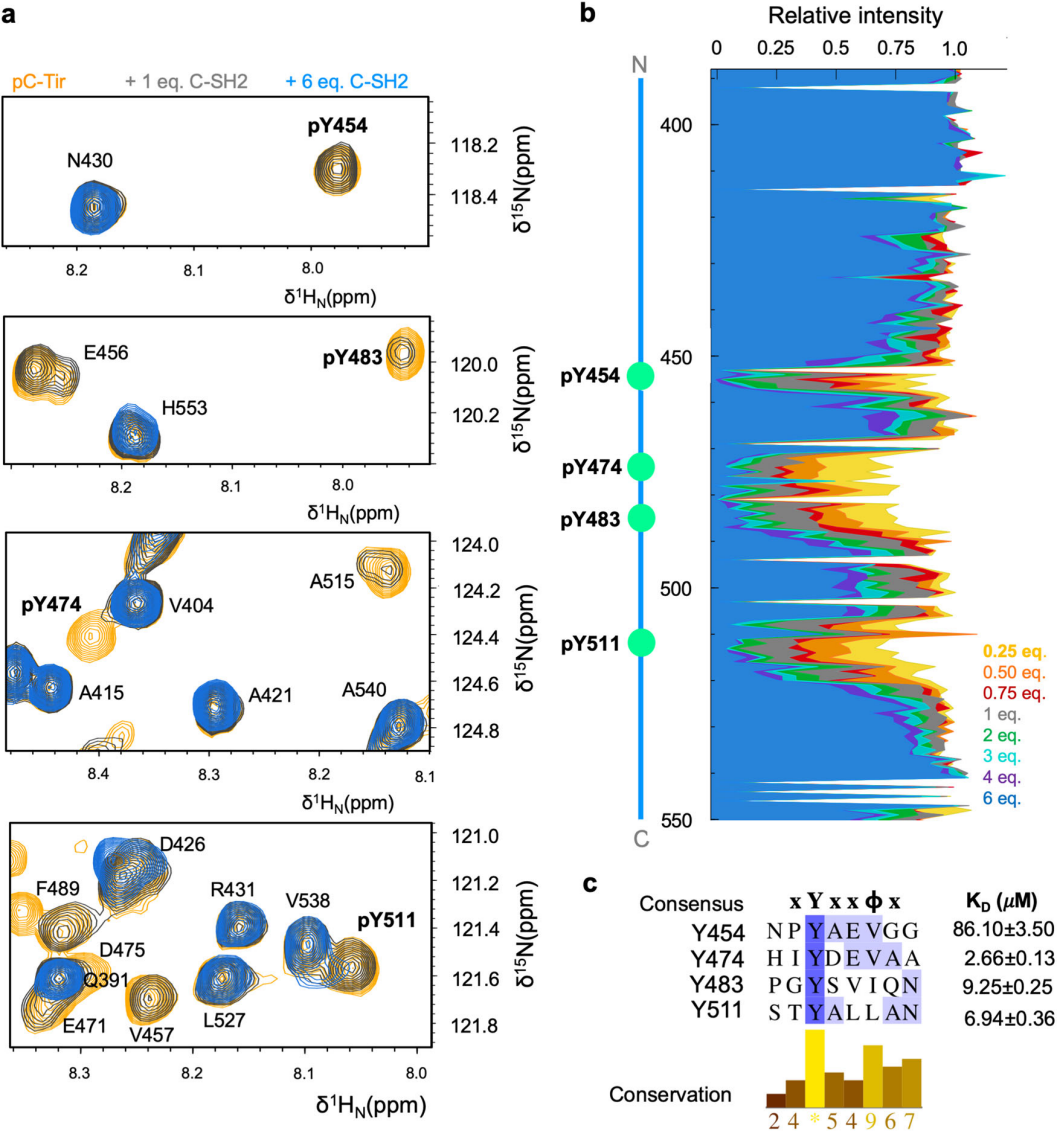
C-Tir contains multiple phosphorylated docking sites. **a** NMR-detected binding of C-SH2 to phosphotyrosine pY454, pY474, pY483, and pY511 before (orange) and after adding 1.0 and 6.0 equivalents of C-SH2 (blue and grey, respectively). **b** Site-specific NMR signal attenuation on pC-Tir due to C-SH2 binding, with local intensity drop around each phosphorylation site. **c** Sequence alignment of xYx(x)ϕ motifs of C-Tir interacting with C-SH2 and their respective apparent K_D_ values obtained by global fitting 1:1 model the intensities drop around each phosphorylation site. Darker shades of blue refer to higher sequence identity. The conservation of physicochemical properties in each alignment position is reported in the corresponding bar-plot below the alignment. Green circles denote phosphorylation tyrosine-sites.

To evaluate the interaction of pC-Tir with an SH2 domain, we titrated unlabeled C-SH2 into a ^15^N-labelled pC-Tir solution and monitored the binding interaction broadening of signals in NMR [^15^N-^1^H^N^]-HSQC spectra (Fig. 7a, b). With the resonance re-assignment of pC-Tir, we identified that upon phosphorylation, all tyrosine sites interact with C-SH2, and not exclusively the Y511-based motif as observed for unphosphorylated C-Tir. The interaction caused a drop in peak intensities around each site (i.e., i = pY454, pY474, pY483, pY511), including roughly residues i + 4 and i-1, even at sub-stoichiometric conditions (Fig. 7b). This suggests a “fuzzy” multivalent binding of C-SH2 to four xYx(x)ϕ motifs, where ϕ is hydrophobic (V/L/I) (Fig. 7c), with multiple pYs on C-Tir interacting with C-SH2 in dynamic equilibrium^73,74^. Except for these residues, backbone chemical shift changes were relatively small, with spectra displaying sharp line widths and low dispersion, both characteristics of a disordered protein. Thus, pC-Tir binds C-SH2 retaining its high level of disorder. The relative signal intensities at each phospho-tyrosine (pY) and surrounding residues approximately reflect the fraction of unbound sites, allowing us to estimate their apparent local binding affinity (Supplementary Figure 13). Phosphorylation enhanced the binding to Y511 site by ~10 fold and enabled other tyrosine-based motifs to engage C-SH2. Among the four pYs, the resonances around pY454 were less broadened during the titration, suggesting lower local binding. The remaining pYs bind C-SH2 with similar strength (~3-9 μM). Overall, phosphorylation at multiple tyrosine sites provides various SH2 docking sites and reinforces the role of Tir as a scaffolding hub.

### Disordered C-Tir binds lipids

Remarkably, Tir ITIMs and cytoplasmic tyrosine-based sequence motifs of host receptors share the ability to adopt α-helical conformations. Indeed, host receptor tyrosine-based motifs can form dynamic/transient α-helices, whether free^75,76^ or membrane-bound state^77^. Their dynamic binding to membranes is postulated to regulate tyrosine sites’ access to phosphorylation, providing a switch between functional and non-functional conformations^78^. The helical conformation in ITIMs/ITAMs is stabilised in vitro in the presence of TFE or through binding to detergent micelles and lipids^76,77,79^. In the presence of TFE, we found that C-Tir exhibited a substantial increase in secondary structure, with the CD signature of a partial helical protein (Supplementary Figure 14a). This conformational change is followed by chemical shift differences (Supplementary Figure 14b**)** and a differential decrease in peak intensities of the C-Tir amide proton resonances in increasing amounts of TFE, mainly around Y511 and Y483 and not at S434 (Supplementary Figure 14c). This effect supports an enhanced α-helical conformation propensity around the two tyrosines, and a putative role in membrane-binding, as postulated for some host tyrosine-based motifs that undergo TFE-induced structural changes^76^.

The ability to form α-helices stabilised by TFE prompted us to investigate lipid binding by C-Tir. We incubated ^15^N-labelled C-Tir with different bicelles, small planar bilayers of long-chain lipids closed by curved micelle-like walls of short-chain lipids. These disk-like structures are membrane models extensively used to explore protein-membrane interactions, including those involving disordered proteins^80^. As long-chain lipids, we used 1,2-dimyristoyl-*sn*-glycero-3-phosphocholine (DMPC) or the acidic lipid 1,2-dimyristoyl-*sn*-glycero-3-phosphorylglycerol (DMPG) mixed with the short-chain lipid 1,2-dihexanoyl-*sn*-glycero-3-phosphocholine (DHPC), offering the possibility to create bicelles with different lipid charge density.

**Fig. 8:**
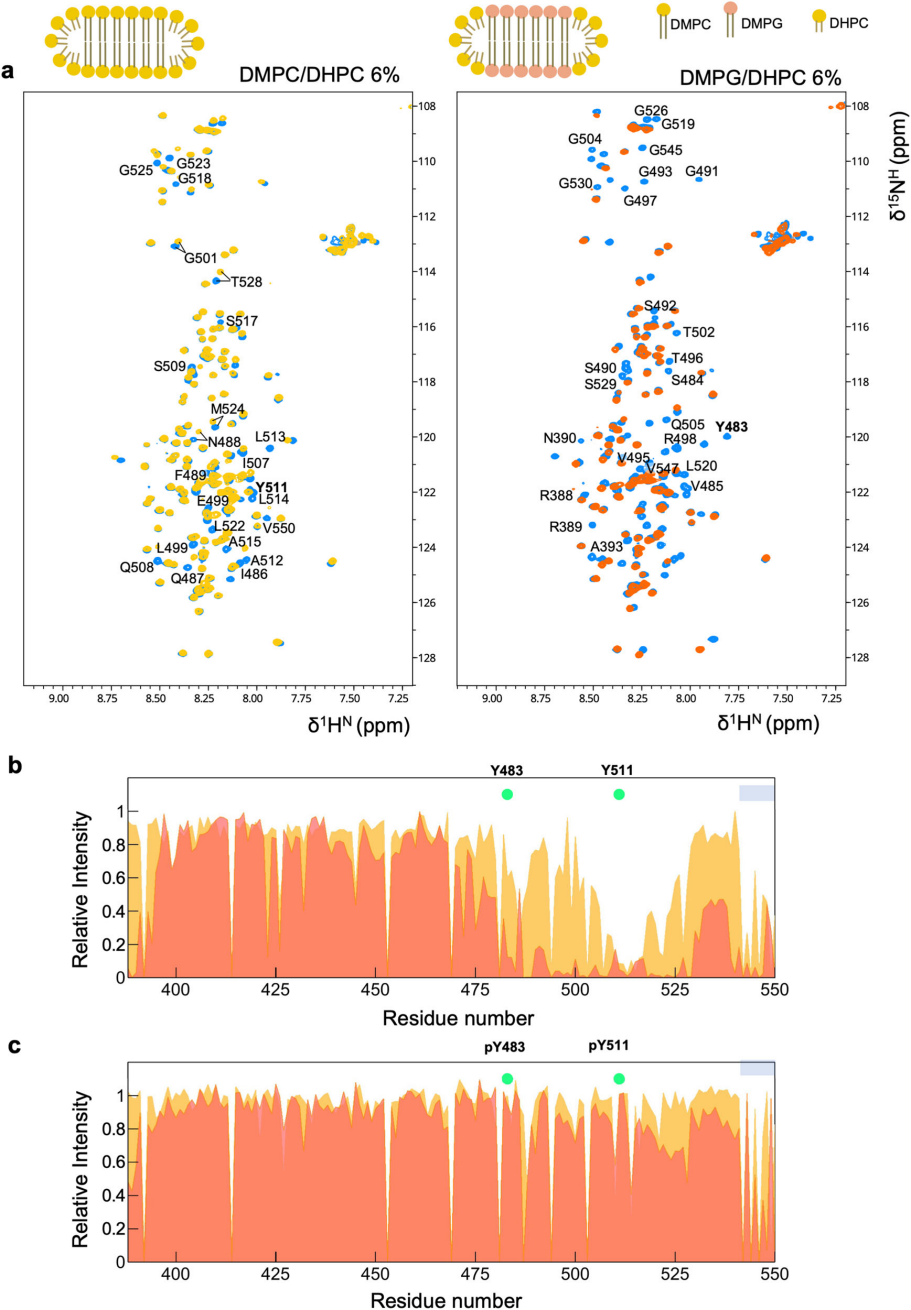
Lipid-binding by disordered C-Tir. **a** NMR [^15^N-^1^H^N^]-HSQC spectra of C-Tir in the absence (blue) and the presence of DMPC/DHPC (6% w/v), yellow) or DMPG/DHPC (6% w/v), orange) bicelles. C-Tir’s HSQC data in bicelles superimpose well to free C-Tir, with identical chemical shifts for most visible resonances, pointing to intrinsic disorder. Residues with NMR signal attenuation upon the addition of DMPC/DHPC bicelles are indicated in the spectra (left). The additional residues affected by DMPG/DHPC are shown (right). **b**, **c** Lipid-induced NMR attenuation profiles are plotted against C-Tir primary sequence. **b** Residues comprising the Y483 and Y511 ITIM motifs became less intense with bicelles, reinforcing their inherent ability to bind lipids. (C) With phosphorylation, the NMR signal attenuation around Y483 and Y511 becomes essentially less pronounced. The grey bar highlights the C-terminal residues affected by lipids.

In the presence of bicelles containing DMPC lipids, we observed a marked residue-specific decrease in the ratio of NMR signals around Y511, yet more subtle, also around Y483 (Fig. 8). This finding shows that C-Tir does interact with non-charged lipid bilayers predominantly via its Y511-based motif, including mostly hydrophobic residues, such as A_512_LLA_515_. We further tested the influence of increasing negatively charged lipid head groups on C-Tir membrane association with bicelles containing DMPG lipids. Our NMR data show a more extended NMR signal attenuation in the presence of DMPG/DHPC bicelles, even including the first stretch of residues of this construct, which display some positively charged residues (e.g., R388 and R389) (Fig. 8). In the context of the full-length protein, these residues define the membrane-proximal region (Fig. 1e, Supplementary figure 3). Given its proximity to the cell plasma membrane, it is reasonable to assume its involvement in membrane binding. Aside from Y511 surrounding residues, the sequence around Y483 is also notably affected by anionic lipid content, indicating that lipid-charge content modulates C-Tir membrane binding modes. Contrary, the residues 395-475 did not show any membrane interaction. This region has a net negative charge (pI~4.5), whereas the sequences displaying membrane ability are positive (pI~9.9), thus in line with an electrostatic model. In this residue range, C-Tir bears two experimental confirmed host protein binding sites: a) NCK Src Homology 2 (SH2) domain binding motif^30^; and b) NPY motif that interacts with the I-BAR domain of host IRSp53/IRTKS^81^. With both bicelles, the last C-terminal residues of C-Tir also show NMR-signal attenuation indicative of membrane-binding (Fig. 8b). These residues A_541_PTPGPVRFV_550_ emerge as part of a potential new lipid-binding region, also affected by TFE (Supplementary Figure 14) and showing pre-existing compaction (Fig. 5). This indicates that C-Tir can undergo multivalent and tunable electrostatic interaction with lipid bilayers via pre-structured elements, suggesting that membrane-protein interplay at the intracellular side might control the activity and interactions of Tir in host cells.

We also tested if phosphorylation of Y483 and Y511 could affect lipid-binding. To this end, we mutated Y454 and Y474 to phenylalanine (Supplementary Table 6) and phosphorylated the remaining two tyrosines. Next, we incubated the phosphorylated and ^15^N-labelled mutant (pC-Tir_454F474F_) with both types of bicelles to probe by NMR lipid binding. Our NMR data reveal that the phosphorylation of Y483 and Y511 drastically hampers the membrane association of those sites (Fig. 8c) while partially unaffecting the ability to interact with anion bicelles of the first and last stretch of residues. Our NMR results show that unphosphorylated C-Tir can adopt pre-formed structures potentially relevant to membrane binding and molecular recognition of signalling proteins.

### Tir-induced actin polymerization and cell death depend on C-Tir lipid-binding

Tir promotes actin polymerization at the bacterial attachment site, leading to pedestal formation^29^; moreover, Tir activates pyroptotic cell death in intestinal epithelial cells^34^. To investigate whether the lipid-binding residues affect the known biological functions of Tir during the infection, we mutated key residues in the C-terminus of Tir (R388, R389, and L513) (Supplementary Table 6). Plasmid-encoded wild-type and mutant Tir were expressed in EPEC-0, which encodes the T3SS and intimin but is missing all the known effector genes, including *tir*^82^. These strains were then used to infect the model intestinal epithelial cell line SNU-C5^34^.

**Fig. 9:**
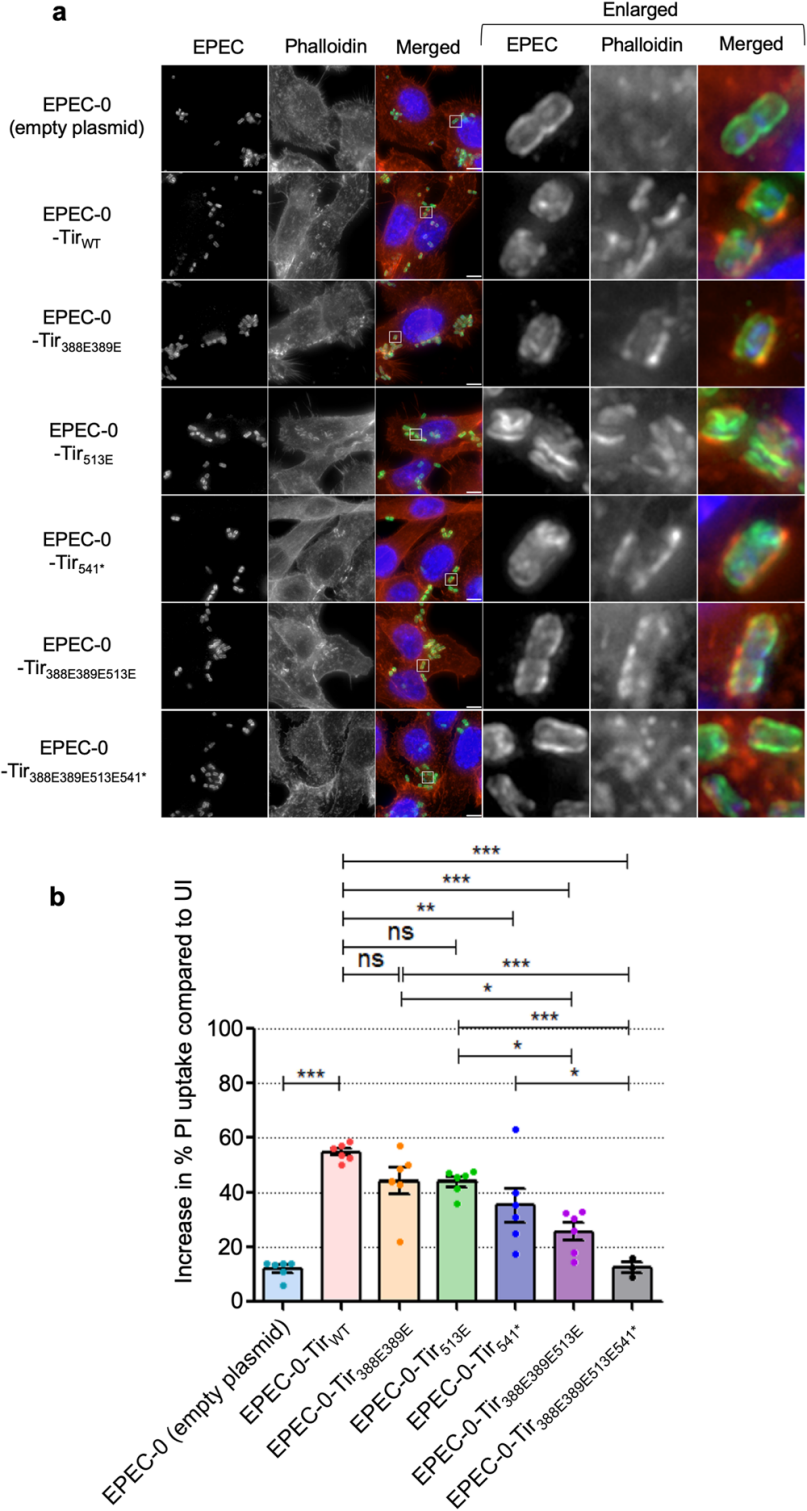
Effect of Tir C-terminus mutations on Tir-induced actin polymerization and cell death. **a** Immunofluorescence images of SNU-C5 cells infected with EPEC-0 (empty plasmid), EPEC-0-Tir_WT_, EPEC-0-Tir_388E389E_, EPEC-0-Tir_513E_, EPEC-0-Tir_541*_, EPEC-0-Tir_388E389E513E_ or EPEC-0-Tir_388E389E513E541*_ at 2 h post-infection. Enlarged cell-attached EPEC are seen in the white box (right-hand side panels). Shown are representative images from 3 independent biological repeats. Scale bar: 5 µm. Blue: DAPI (DNA); Green: EPEC; Red: phalloidin (actin). **b** PI uptake into SNU-C5 cells infected with EPEC-0 (empty plasmid), EPEC-0-Tir_WT_, EPEC-0-Tir_388E389E_, EPEC-0-Tir_513E_, EPEC-0-Tir_541*_, EPEC-0-Tir_388E389E513E_, or EPEC-0-Tir_388E389E513E541*_ at 8 h post-infection. Data shown are mean ± SEM from 3 (EPEC-0-Tir_388E389E513E541*_) or 6 (all other strains) independent biological repeats. Statistical analysis was performed using 1-way ANOVA with Tukey’s post-test. *p ≤ 0.05; **p ≤ 0.01; ***p ≤ 0.001; ns: non-significant.

We first mutated the positively charged residues R388 and R389, as well as L513 located in the Y511 ITIM motif to the negatively charged glutamic acid (Supplementary Table 7). Similar to EPEC-0-Tir_WT_, EPEC-0-Tir_388E389E_ and EPEC-0-Tir_513E_ triggered the formation of actin pedestals (Fig. 9a) and propidium iodide (PI) uptake as a marker of lytic cell death (Fig. 9b). We then introduced a STOP codon at residue A541, removing the ten residues of the C-terminus tail (A_541_PTPGPVRFV_550_), which are also implicated in lipid-binding (Supplementary Table 7), creating EPEC-0-Tir_541*_. While pedestal formation was unaffected in EPEC-0-Tir_541*_-infected cells (Fig. 8a), we detected a mild yet significant reduction in PI uptake compared to EPEC-0-Tir_WT_ (Fig. 9b) that became more evident over time (Supplementary Figure 15). Accordingly, mutations in all three sites individually did not affect pedestal formation and only resulted in a limited reduction in Tir-induced cell death.

Next, we generated a triple R388E / R389E / L513E mutant (Supplementary Table 7). The resulting strain EPEC-0-Tir_388E389E513E_ triggered typical actin pedestals (Fig. 9a); however, we observed significantly reduced cell death compared to both EPEC-0-Tir_388E389E_ and EPEC-0-Tir_513E_ (Fig. 9b). Finally, we mutated all four sites simultaneously, including point mutations and the C-terminus ten residue truncation (Supplementary Table 7). The resulting EPEC-0-Tir_388E389E513E541*_ was impaired in actin pedestal formation (Fig. 9a) and was not cytotoxic, similar to the negative control EPEC-0 with an empty plasmid (Fig. 9b). Therefore, while the lipid-binding sites at the C-terminus of Tir appear to be essential for its biological functions, they might be partly redundant as only the quadruple mutant affected Tir-induced cell death and pedestal formation.

## Discussion

Although IDPs represent only 5% of typical bacterial proteomes, it has become clear that they control critical aspects of bacterial biology^83^. Here, we have determined the structural disorder propensity of EPEC O127:H6 sequences and two additional representatives of A/E bacteria: EHEC O157:H7 and CR ICC168. We observed that the three studied prototype strains of A/E pathogens have a structurally diverse repertoire of protein effectors enriched in disorder-prone residues. Some effectors are fully disordered proteins with a high density of host-like short linear motifs, challenging the general trend that prokaryotic proteins are less disordered. Among such disordered effectors emerged Tir, a 56 kDa cell surface receptor able to reshape host cellular behaviour during infection. We examined the structure of Tir from EPEC O127:H6, finding that its intracellular domains (i.e., N-Tir and C-Tir) are flexible with IDR features similar to those found in the cytoplasmic domains of host transmembrane proteins. We unravelled that the N-Tir is highly disordered (~52%) but forms a stable antiparallel dimer. The central region of N-Tir comprises an SBB domain followed by a coiled-coil dimerization helix. The structural and functional implications of an OB-fold in Tir are still unknown. But, many SBB-containing proteins with OD-fold often act as modules that bind to various RNAs, DNAs, phospholipids, and other proteins^56^. Many OB-folds are domains in proteins that frequently oligomerize. Clustering of Tir triggers host signalling events when binding to intimin. The extracellular intimin binding domain (IBD) of Tir binds intimin as a dimer^35^ in a reticulating model^84^. Thus, Tir’s intracellular self-assembly of SBB domains questions whether host signalling activation involves intimin-induced dimerization or structural changes of preformed dimers. It would be interesting to assess the impact of N-Tir oligomerization on the function of Tir. Nevertheless, the N-terminus Tir dimerization embodies a typical feature of cell receptors that Tir is meant to mimic.

Moreover, NMR analysis showed that unbound C-Tir is not exclusively disordered but can adopt partial secondary structural elements and long-range compaction around phosphorylation sites and residues involved in biomolecular interactions (*i.e*., host protein and lipid-binding). This observation supports the idea of transient pre-structured motifs (PreSMos) as potential pre-existing signatures for binding and function within target-free Tir^85^. However, we cannot rule out that C-Tir also undergoes structural rearrangement upon binding via induced fit^86^.

As in disordered cytosolic tails of host receptors, C-Tir displays tyrosine-based motifs, which are phosphorylated by Src family PTKs enabling the interaction with SH2–containing proteins. Interestingly in C-Tir, we found that the motif around Y511 can bind a single C-SH2 without phosphorylation. NMR shows that the residues adjacent to Y511 define a binding site for C-SH2, likely serving as a precursor to a tighter binding upon phosphorylation. Another notable feature of this motif is its α-helical propensity. Although we cannot tell whether a helix is a prerequisite for C-SH2 binding, our results suggest an alternative interaction mode to the “two-pronged plug two-hole socket” model^87,88^. This atypical SH2 binding may offer an additional mechanism for engaging with host SH2-containing proteins pre-phosphorylation. The Y511 motif also interacts with lipid bilayers. Notably, subsequent multi-site phosphorylation of C-Tir enables the transition from binary to multivalent fuzzy complex with C-SH2. These observations highlight a functional diversity for C-Tir and possible cooperation between host proteins, phosphorylation, and the membrane in signalling processes inside the host^14^.

Some T-cell receptor cytoplasmic disordered domains associate with the plasma membrane via tyrosine-based motifs with a helical propensity, and such association regulates phosphorylation and downstream signalling^78^. Like the CD3ɛ cytoplasmic domain of the T-cell receptor^77^, C-Tir also has two tyrosines involved in lipid-binding and helical propensity. Our results suggest that the C-terminal half Tir can mimic this regulatory stratagem to fine-tune its ability to interact with human cell components, thereby interfering with normal cellular functions. Further studies are needed to corroborate this initial observation. Nevertheless, we show that the C-Tir can interact with membranes via two ITIMs and its last C-terminal residues. Importantly, our results establish that this membrane affinity is residue-specific and modulated by lipid composition and phosphorylation in a quantitative and site-resolved way, thus suggesting the existence of a regulation layer based on lipid composition and phosphorylation. Our results support that transiently structured lipid-binding regions might bury tyrosine residues rendering them inaccessible, while their phosphorylation perturbs membrane anchoring^89^. Importantly, we uncover that lipid binding by Tir C-terminus is essential for Tir-induced host cell death and pedestal formation. Interestingly, Tir-induced cell death was previously shown to be dependent on mechanosensitive Ca2+ channels and can be modulated by membrane mechanical stress^90^. As high density of lipid-binding proteins, such as in the case of intimin-clustered Tir, can potentially produce lateral pressure to bend the lipid membrane^91^, it remains to be seen whether the lipid-binding ability of Tir directly results in the mechanical changes in the plasma membrane, leading to cell death.

The flexibility and conformational plasticity of C-Tir make it readily accessible for phosphorylation. We found in C-Tir four bona fide tyrosine phosphorylation sites modified by Fyn that render the ability for multivalent/promiscuous binding, supporting the idea of Tir acting as a signalling hub. Even though we only used protein fragments and explored one phosphorylation state, our results support the possibility of four phospho-tyrosines (pY) in the C-Tir sequence coexisting simultaneously. All can bind the C-SH2 in the micromolar range, as reported for other pY-containing motifs^92^. Interestingly, the pY454 site, which displays the lowest affinity, is part of a conserved NPY binding motif for the I-BAR domain of host IRSp53/IRTKS, linking Tir to the actin polymerization machinery^81^ in a phosphorylation-independent manner. So, Y454 might act as a binding site for distinct and competing host proteins depending on its phosphorylation state. The superposition of interplaying functional elements observed in C-Tir mimics the mechanics of human intrinsically disordered domains. Future work using high-resolution structural studies targeting the interaction with multiple partners and different C-Tir’s phosphorylation states will provide additional insights into Tir binding specificity and action mode.

In brief, we establish an updated picture of Tir’s intracellular side, supporting that in host cells, Tir dimers can interact with the host plasma membrane via their C-terminus with implications to downstream signalling triggering actin polymerization and cell death. Phosphorylation impacts lipid binding and renders the ability to interact with multiple host SH2 domains. In eukaryotic cells, protein disorder is a frequent feature of protein hubs controlling the complex intracellular networks that influence physiological responses^4,93^. Notably, the structural disorder of Tir and several other A/E effectors reinforce the idea of a positive evolutionary selection towards disordered proteins in the pool of secreted effectors by bacterial pathogens to target host cellular machinery^19^. Therefore, ELMs in disordered regions of A/E effectors may play a key structural and signalling role in protein function through interactions with host factors facilitated by their conformational flexibility. Given the ubiquitous presence of IDPs as transcriptional factors^94^ and, more generally, as hubs in host networks^93^, bacterial effectors with host-like disordered protein features are an efficient way to subvert host eukaryotic systems and promote infection.

## Methods

### Disorder prediction and short linear motif analysis

We collected the sequence of A/E pathogen effectors and corresponding reference proteomes from the UniprotKB database^95^ and a representative set of 20365 human proteins from the human reference proteome (Supplementary Table 1). For A/E bacteria, we assembled three sets of effectors for the following representative strains: CR (strain ICC168), EHEC O157:H7, and EPEC O127:H6 with 28, 39, and 24 sequences, respectively. To compute disorder propensity at the residue level, we used the structural disorder predictors DISOPRED 3^40^ and IUPred 1.0 (set to “long” mode)^41^. Disordered residues were those with a propensity score equal to or above 0.5. We used this metric to calculate the fraction of disordered residues for each protein. Next, we used the one-sided Mann-Whitney U-test^96^ to compare the disorder fraction distribution of effector collections and their corresponding proteomes. Only effectors with reference proteomes were considered for disorder fractions comparison and removed from their proteome sets. Based on per-residue scores and aggregated disorder fractions, we classify each protein according to the structural categories adapted from^97^ (IDP: Intrinsically disordered proteins; PDR: Proteins with intrinsically disordered regions; FRAG: Proteins with fragmented-disorder; NDR: Not disordered proteins; ORD: Ordered Proteins). For this classification (Supplementary Table 2), we changed the original threshold for disordered stretches from 30 amino acids to 22 to be more in line with the shorter average length of prokaryotic proteins compared to eukaryotic ones (320/472 = 0.68)^98^. We downloaded the list of eukaryotic short linear motifs (ELMs) from the ELM database^99^ (version 1.4, May 2017). Next, we searched for putative ELMs in effector sequences using the ANCHOR tool^100^. We calculated the motif density for each effector sequence as the fraction between the amino acids belonging to putative ELMs predicted as disordered and the total number of amino acids predicted as disordered. See all data in Supplementary Table 3.

### Protein expression and purification

The genes encoding for N-, NS-, SBB- and C-Tir, (residues 1–233, 61–200, 76–180, and 388–550, respectively; Supplementary Table 4) were amplified by PCR from a synthetic *E. coli* codon-optimised gene of the full-length protein (Uniprot code B7UM99 | TIR_ECO27). The human SHP-1 C-SH2 domain (residues 101-217; Uniprot code P29350 | PTN6_HUMAN) was PCR-amplified from a vector with the cDNA encoding the tandem SH2 domains of SHP-1 acquired from Addgene (pGEX SHP-1(NC)-SH2; Plasmid #46496). We inserted all PCR products into the IPTG-inducible pHTP8 vector (NZYTech) fused to an N-terminal Trx-His_6_-tag and a C-terminal Strep-tag. These constructs encoded TEV or HRV-3C protease recognition sites to remove the Trx-His_6_-tag linked to N-, NS- and SBB-Tir, or C-Tir and C-SH2, respectively. For C-Tir, Y454 and Y474 were mutated to phenylalanine using primers in Supplementary Table 6, resulting in the construct C-Tir_F454F474_.

*E. coli* BL21 Star (DE3) pLysS were transformed with the desired construct and grown at 37 °C to a mid-log phase (OD_600_ ~ 0.6–0.8) in LB media containing kanamycin (50 μg/mL) and chloramphenicol (34 μg/mL). Protein expression was induced at 20 °C with 0.1 mM and 1 mM IPTG for SHP-1 C-SH2 domain and Tir constructs, respectively. After 24 h, bacteria were pelleted at 4 °C by centrifugation and stored at −20 °C until purification. For N- and NS-Tir and C-SH2, frozen cell pellets were resuspended in lysis buffer A (50 mM Tris-HCl, 150 mM NaCl, 1 mM EDTA, 1 mM DTT, pH 7.5) supplemented with EDTA-free protease inhibitor cocktail; and then lysed in a French Press. IDPs are known to resist high temperatures. The cell pellets with C-Tir were resuspended in lysis buffer B (50 mM Tris-HCl, 150 mM NaCl, 5 mM EDTA, 1 mM PMSF, pH 7.5) and lysed by boiling at 90 °C for 30 min in a water bath^101^. Total cell lysates were clarified by centrifugation for 40 min, 42000 rpm and 4 °C. For C-Tir, the resulting pellet was washed and resuspended two times in buffer B with 1% Triton X-100 and 0.5 M urea. The supernatants containing the soluble proteins were loaded onto 5 mL Strep-Tactin®XT high-capacity columns (IBA Lifesciences) equilibrated in buffer A. The N-terminal Trx-His_6_ tag, in C-Tir or C-SH2, was cleaved on-column with HRV-3C protease (1:100 protease:target protein ratio) overnight at 4 °C. We dismissed this step for N-Tir variants since they were expressed in cells containing an auxiliary plasmid, pRK793 (Addgene), which produces MBP-fused TEV protease S219V mutant. This approach enabled in vivo Trx-His_6_-cleaved N-Tir variants with a C-terminal Strep-tag^102^. Proteins were eluted with the buffer D (100 mM Tris-HCl, 150 mM NaCl, 1 mM DTT, 1 mM EDTA, pH = 8.0) containing 50 mM Biotin. Pure fractions were pooled and purified using a Superdex 200 Increase 10/300 (GE Healthcare). Pure C-Tir was eluted in 20 mM sodium phosphate, 150 mM NaCl, 1 mM EDTA pH 6.5; C-SH2 in 20 mM HEPES, 150 mM NaCl, 1 mM DTT, pH = 6.8; and pure N-Tir, NS-Tir and SBB in 20 mM HEPES, 150 mM NaCl, 1 mM EDTA, 1 mM TCEP, pH=6.5.

### (un)Labelling for NMR experiments

For producing uniformly isotopic-labelled proteins, cells in LB were transferred before induction to M9 minimal media containing stable isotope precursors sources: 1 g/L of ^15^N-NH_4_Cl (U-^15^N-labelling) or 1 g/L of ^15^N-NH_4_Cl together with 2 g/L of ^13^C D-glucose (U-^13^C/^15^N-labelling). We also used reverse labelling to help overcome the signal-overlap problem often found in IDPs spectra. To this end, we produced isotopic-labelled C-Tir with a small set of unlabeled residues by growing cells in M9 media containing 1 g/L of ^15^N-NH_4_Cl and 1 g/L of unlabeled amino acids supplemented exogenously 1 hour before induction. We selected unlabeled amino acids with reduced ^14^N-isotope scrambling (i.e., L-arginine, L-asparagine, L-glutamine, or L-histidine)^62^ to avoid unwanted reverse labelling. So, only the supplemented unlabeled amino acids were selectively absent from NMR spectra of C-Tir. For paramagnetic NMR experiments, single cysteine variants of C-Tir were produced as described above and labelled with 1-oxyl-2,2,5,5-tetramethyl-3-pyrroline-3-methyl)-methanethiosulfonate (MTSL). A 10-fold excess of MTSL was immediately added to DTT-free samples after elution from PD-10 columns and left reacting overnight at 4 °C in the dark. Excess MTSL was removed using PD-10 columns, and samples were exchanged into the NMR buffer.

### NMR spectroscopy

We recorded all NMR experiments on a Bruker Avance II+ spectrometer operating at 18.8 T (800 MHz) and equipped with a helium cold TXI-cryoprobe. Spectra were processed with TopSpin and NMRPipe^103^ and analysed using CARA^104^. ^1^H chemical shifts were referenced directly, and ^15^N chemical shifts indirectly using 3-trimethylsilyl-1-propanesulfonic acid sodium salt (DSS; methyl ^1^H signal at 0.00 ppm).

For C-Tir’s backbone resonance assignment, we recorded a set of 3D HNCO, HN(CA)CO, HNCA, HN(CO)CA, HNCACB, HN(CO)CACB, together with 2D [^15^N-^1^H^N^]-HSQC experiments at 283 K using 250-950 μM U-^13^C/^15^N-labelled samples in 20 mM sodium phosphate, 150 mM NaCl, 1 mM EDTA pH 6.5 with 20 μM DSS and 8% ^2^H_2_O (NMR buffer). We also measured 3D [^1^H,^15^N]-TOCSY-HSQC and [^1^H,^15^N]-NOESY-HSQC experiments using a 480 μM U-^15^N-labelled sample. In reverse labelling experiments with unlabelled L-arginine, L-asparagine, L-histidine, or L-glutamine residues, we recorded four regular 2D [^15^N-^1^H^N^] HSQC spectra. By comparing 2D [^15^N-^1^H^N^]-HSQC data of the uniformly labelled C-Tir with spectra where only one amino acid type is unlabeled, we could link the missing NMR signals to those respective amino acids. We used this helpful information to efficiently identify C-Tir’s backbone resonances and validate the sequential NMR assignment. This reverse labelling approach helped identify resonances by amino-acid type and simplified the spectral overlap by reducing observed peaks without any disadvantages to the protein expression and should be readily applied to other IDPs. Using the experimentally assigned chemical shifts, we employed the nsSPC^67^ and CheSPI^68^ methods to provide local structure information within the disordered C-Tir.

The resonances of backbone nuclei of N-Tir disordered residues were assigned using standard triple-resonance spectra. We collected HNCA, HN(CO)CA, HNCACB, HN(CO)CACB, HNCO, and HN(CA)CO experiments at 278 K using 600-760 μM U-^13^C/^15^N-labelled N-Tir samples in 20 mM HEPES, 150 mM NaCl, 1 mM EDTA, 1 mM TCEP, pH=6.5 with 8% ^2^H_2_O, and 20 μM DSS. Comparison of the [^15^N-^1^H^N^]-HSQC data of N-Tir to NS-Tir spectra enabled us to assign and corroborate the resonances within the flanking regions. Due to their distinct hydrodynamic properties, variable-temperature NMR experiments often report differently folded and disordered residues. Accordingly, we collected NMR at different temperatures from 5 to 35 °C, exploiting the differential temperature-dependent NMR sensitivity to ordered and disordered regions to investigate the N-Tir protein order-disorder interplay. We have probed mainly the N-Tir’s flexible protein regions with NMR at low temperatures, with ordered residues’ resonances broadened beyond detection due to slower tumbling. The folded residues’ resonances then became observable due to faster tumbling with higher temperatures. In contrast, the exchange of amide protons with the solvent at increased temperatures led to broadening of exposed disordered residues’ resonances, only allowing the detection of folded and less solvent-exposed regions. In addition, protein backbone resonances of NS-Tir were assigned from Best-TROSY-based 3D spectra^105^ [HNCO, HN(CA)CO, HNCA, iHNCA, HN(CO)CA, HNCACB, HN(CO)CACB, HNHA, H(CCO)NH and CC(CO)NH] all recorded with nonuniform sampling on a 1.23 mM U-[^15^N,^13^C]-labelled protein in the NMR buffer at 278 K. We also measured a 3D [^1^H,^15^N]-TOCSY-HSQC and [^1^H,^15^N]-NOESY-HSQC with a mixing time of 120 ms using a 607 μM U-^15^N-labelled sample. We computed the secondary structure propensities in terms of α-helix or β-sheet from the assigned chemical shifts using N-TALOS.

### Chemical shift perturbations

NMR is highly sensitive to probe changes in the chemical environment. We extensively used this method to detect subtle chemical perturbations due to 2,2,2-Trifluoroethanol (TFE)-induced structural changes, lipid or protein binding, and tyrosine phosphorylation. To monitor the effect of TFE on C-Tir, we prepared protein samples (100 μM) in the NMR buffer with increasing amounts of TFE (1, 2, 5, 10 and 12.5% (v/v)) and acquired a 2D [^15^N-^1^H^N^]-HSQC spectrum for each condition. TFE-induced effects were determined as the ratio of [^15^N-^1^H^N^]-HSQC cross-peak intensity of the samples in the absence and presence of TFE.

### ^15^N-*R*_1_ and *R*_2_ experiments

T1 and T2 relaxation measurements were obtained using standard pulse sequences as previously described^106^ at 283 K. For the T1 relaxation experiments, the time intervals for inversion recovery were set at 20, 60, 100, 200, 400, 600, 800, 1200, 1400, 1600, 1800 and 2000 ms. For T2, the number of loops were set at 1, 2, 4, 8, 10, 12, 14, 16, 20, 24, 30, 40. Regarding the Free Induction Decay (FID) parameters, the data matrix was established with (^1^H)1024 × (^15^N) 256 points and 16 scans for all conducted experiments. Finally, the relaxation rates were obtained by applying an exponential function to the peak intensities as implemented in CcpNmr AnalysisAssign^107^.

### Paramagnetic relaxation enhancement experiments (PREs)

We acquired PRE datasets for five paramagnetic centres engineered at different positions on the C-Tir to probe long-range contacts. Those positions were evenly scattered around the C-Tir sequence and preferable at serine residues in the wild-type. ^1^H-^15^N-HSQC spectra were acquired for the paramagnetic and diamagnetic (reduced with ascorbic acid) states C-Tir. Peak intensities of the paramagnetic (*I*_para_) and the diamagnetic (*I*_dia_) states were extracted by fitting the peaks with Lorentzian line-shapes. Only well-resolved resonances were used to quantify PRE ratios. Simulated random coil PRE values were calculated from a pool of 10,000 conformers of C-Tir using the average line width in the ^1^H dimension of the diamagnetic spectrum of each protein. ΔPREs were calculated as the difference between simulated and experimental (I_para_/I_dia_) data for each residue and smoothed with a 1D Gaussian kernel of window size = 7 and one standard deviation, interpolating the values for prolines or missing signals used to generate the heatmaps^108^.

### Small-angle X-ray scattering (SAXS)

We employed synchrotron SAXS coupled with size exclusion chromatography (SEC) to probe the overall size and conformational properties of Tir intracellular regions and their ab initio shape (N-Tir) or ensemble (NS-Tir and C-Tir) representations. SAXS data for C-Tir were collected on the BM29 beamline (ESRF, Grenoble, France) and for N- and NS-Tir in the B21 beamline (DSL, Didcot, UK)^109^, exploiting their unique in-line HPLC systems (Shimadzu and Agilent 1200 respectively). We injected 50 μL samples with 10.0–25.0 mg mL^−1^ of SEC-purified protein into a 2.4 mL Superdex 200 Increase 3.2/300 column (GE) at a flow rate of 0.075 mL min^−1^ at BM29 and into a 4.6 mL Shodex KW403-4F column at a flow rate of 0.16 mL min^−1^ at B21. We acquired 1-second and 2-second frames in BM29 and B21, respectively. We did not detect radiation damage or signs of interparticle interference or aggregation. The SEC mobile phase consisted of 20 mM Phosphate pH 6.5, 150 mM NaCl, and 1 mM EDTA, for C-Tir, and 100 mM Tris·HCl at pH 8.0, 150 mM NaCl, 2 mM DTT, and 1 mM EDTA for N-Tir and NS-Tir. The scattering intensities from the respective monomeric (C-Tir) or dimeric elution (N-Tir or NS-Tir) single-peak region were integrated and buffer subtracted to produce the SAXS-profiles using ScÅtter (http://www.bioisis.net) and further processed with ATSAS^110^. A 50 µL series of samples with 1, 2.5, 5, 7.5, and 10 mg/ml were also run in batch mode. The scattering intensities of the 7.5 and 10 mg/mL injections of C-Tir (10 frames each) were merged to produce a SAXS profile. The *R*_*g*_ values were estimated using the Guinier approximation in the range *s* < 1.3/*R*_*g*_. From SEC-SAXS, we created low-resolution ab initio molecular envelopes for N-Tir and NS-Tir, with the program DAMMIF and GASBOR in the ATSAS package^110^, assuming point symmetry P2. With DAMMIF, twenty independent models were generated, superimposed, and averaged to define the models’ most populated volume and assess modelling robustness. Details are in Supplementary Table 5.

### Structural ensembles

We used AlphaFold2-multimer^39^, a refined version of AlphaFold2^52^ for complex prediction, to predict the structure of the folded part of the NS-Tir dimer. We did not use template structures in the modelling, iterated for up to 48 recycles, followed by energy refinement with AMBER using default settings implemented in LocalColabFold^111^ and using MMseqs2 for creating multiple sequence alignments^112^. We assessed the AF-2 models’ confidence by the predicted Local Distance Difference Test (pLDDT) and inter-chain complex Predicted Alignment Error (PAE), i.e., the uncertainty about the interface. pLDDT is closely related to the pre-existing metric lDDT-Cα^113^ that measures the local accuracy of a prediction by determining the fraction of preserved local distances (higher is better). As a superposition-free method, lDDT is insensitive to relative domain orientation and correctly identifies segments in the full-length model deviating from the reference structure. pLDDT is given on a scale from 0-100. Regions with pLDDT > 90 are expected to have high accuracy. Parts displaying low pLDDT and high disorder propensity that flanked the folded dimer were trimmed from the AF-2 model and modelled as an ensemble. Despite the impressive performance of AF2 algorithms in predicting the native single static lowest energy structure of ordered proteins, they fail to represent disordered regions accurately^114^. Thus, the disordered regions of NS-Tir (61 to 96 and 187-200) were built on the predicted model using the statistical coil algorithm Flexible-Meccano (FM)^53^. Ten thousand conformations were individually compared to experimental SAXS data using EOM^115^ to find those subsets of structures that collectively best fit the SAXS data.

For C-Tir ensemble modelling, we also used the EOM approach to select a subset of conformations from a large starting pool in simultaneous agreement with intramolecular distances from PREs and overall size/shape distribution encoded by the SAXS data. In brief, we created 50000 structures with FM^53^, followed by side-chain modelling^116^, energy-refinement and in-silico incorporation of multiple spin-label dispositions at single-cysteines to interpret the PRE data^117,118^. We simulated the SAXS pattern for each conformer using CRYSOL^110^ and back-calculated the intramolecular ^1^H-^15^N-PRE-PRE rates assuming the Solomon-Bloembergen approximation^117^. We used multiple states derived from a 100 ns Molecular Dynamics (MD) simulation^118^. We extracted a frame every 100 ps to create a library of 1000 different MTSL conformations used to *in-silico* label each single-cysteine residue C-Tir conformer with multiple sterically allowed spin-label dispositions. This MTSL ensemble representation enabled the estimation of the order parameters associated with the dipolar proton-electron interaction vector motion^119^. With this strategy, we independently calculated the PRE rates for V405C, S428C, S463C, S509C, and S536C. We next searched for subsets of 200 models that best reproduce the five PRE rates datasets and the experimental SAXS profile using a genetic algorithm^115^.

### AlphaFold-2 modelling of unphosphorylated C-Tir bound to C-SH2

Again, we used AlphaFold-Multimer to predict the binding interface as described above for NS-Tir. As a first stage, we used the sequences of C-SH2 and C-Tir as input to predict the 3D structure of C-Tir bound to C-SH2. Next, we run independent predictions replacing the full-length C-Tir with the ITIM sequence of C-Tir mapped to bind C-SH2, comprising the G_506_IQ**STYALLA**NSGG_519_ peptide. As before, model confidence was assessed by the pLDDT and PAE metrics. PAE is not an inter-residue distance map or a contact map but the expected distance error in Å. It indicates the expected positional error at residue x if the predicted and actual structures are aligned on residue y (using the C, N and C atoms). PAEs are measured in Å and capped at 30 Å. PAE helps to assess the confidence in the relative position and orientation of the model parts (e.g., two domains or chains). For residues x and y in two chains, if the PAE values (x, y) are low, AF-2 predicts the chains to have well-defined relative positions and orientations^120^.

### CD spectroscopy

We performed Far-UV CD spectroscopy in a J-815 spectrophotometer (Jasco), using a 1-mm optical pathlength cuvette for high performance (QS) (Hellma). The protein concentration was 6, 10, or 11.9 μM for N-Tir, NS-Tir, or C-Tir samples. N-Tir and NS-Tir were in 0.4 mM Tris-HCl pH 8, 15.0 mM NaF, and 0.1 mM TCEP. C-Tir was in 2 mM phosphate pH 6.5, 15 mM NaF and 0.1 mM EDTA supplemented with 0, 5, 10, 20, and 50% TFE. CD spectra are averages of 5-10 accumulations collected in the 190 nm to 260 nm range using a 0.1 nm data pitch and 2 nm bandwidth with a data integration time (DIT) of 8 sec at a scan speed of 50 nm/min at 10 °C. The thermal denaturation of NS-Tir was followed by monitoring temperature-dependent stepwise changes in spectral features ranging from 5 to 95 °C in 5 °C steps. We also recorded the CD signal at 222 nm probing for α-helix secondary structure by applying a temperature ramp of 1 °C min^−1^.

### Tyrosine phosphorylation

Fully phosphorylation of four tyrosine residues in C-Tir was achieved by incubating the protein with human recombinant Fyn Kinase (Thermo Fisher Scientific)^75^. To this end, uniformly ^13^C/^15^N-labelled C-Tir (1.1 mM) was incubated with Fyn (1.93 μM) overnight at 25 °C in 20 mM HEPES pH 7.5 150 mM NaCl, 12 mM MgCl_2_ and 5.5 mM ATP. The amount of Fyn kinase used took into account its optimal specific activity at 25 °C and pH=7.5, and its capacity to completely phosphorylate four potential phosphorylation sites. C-Tir’s full phosphorylation was assessed by comparing 2D [^15^N-^1^H^N^]-HSQC data of pC-Tir to non-phosphorylated protein (969 μM) in the same buffer. Several aliquots of an equivalent ^15^N-labelled C-Tir were collected at different time points (2, 4, 8, 16, 24, 32, 48, and 64 minutes) upon adding Fyn to monitor the reaction. These sample aliquots were incubated at 95 °C for 5 min to stop the phosphorylation reaction. A 2D [^15^N-^1^H^N^]-HSQC spectrum of non-phosphorylated C-Tir was acquired as a control. Spectral dimensions were Ω_1_(^15^N) × Ω_2_(^1^H) = 23.5 ppm × 10.99 ppm, and the acquisition time was 68 ms in the Ω_1_ dimension and 92 ms in the Ω_2_ dimension. 2D [^15^N-^1^H^N^]-HSQC spectra were acquired for the different samples (50 μM each) using the same acquisition parameters as the control samples. The stubble changes in phosphorylated C-Tir (pC-Tir) backbone resonances were checked and re-assigned by HNCACB and HNCO experiments measured for the pC-Tir.

### Phosphorylated C-Tir and binding to SHP-1 C-SH2 domain

Once C-Tir tyrosine phosphorylation was tested and implemented, we produced a ^15^N-labelled pC-Tir sample to reconstruct and investigate C-Tir interaction with the SHP-1 C-SH2 domain. As such, pC-Tir (95 μM) was titrated with 0.25, 0.5, 0.75, 1, 2, 4 and 6 equivalents of unlabeled C-SH2. 2D [^15^N-^1^H^N^]-HSQC spectra were acquired using the same parameters described. Intensity ratio perturbation (${I}_{{obs}}^{i}$) of the NMR signals from pC-Tir were plotted against the molar ratio of C-SH2/C-Tir. Peaks adjacent to each phosphorylation site exhibited a similar intensity drop, so we clustered them by site and globally fitted them to a 1:1 binding model using the equation:

1 $$	{I}_{obs}^{i}={I}_{max}^{i}-({I}_{max}^{i}-{I}_{min}^{i}).\\ 	\frac{({K}_{d}.[CSH2].\,[CTir]).\sqrt{\left(\right.{K}_{D}.[CSH2].[CTir]-4.[CSH2].[CTir]}}{2.[CTir]}$$

where ${I}_{\max }^{i}$ is the maximal relative intensity of protein in free-state and ${I}_{\min }^{i}$ is the minimal relative intensity of bound protein under saturation conditions for residue *i*. ${K}_{D}$ is the apparent dissociation constant for each site. $[{CTir}]$ is the total protein concentration of C-Tir, and $[{CSH}2]$ is the concentration of the C-SH2. ${K}_{D}$ and ${I}_{\min }^{i}$ were defined as adjustable parameters. We employed the same approach to estimate the affinity of unphosphorylated C-Tir to C-SH2, by collectively fitting Eq. 1 to the NMR attenuation of residues A_512_LLA_515_.

### Bicelles preparation

Bicelles with different charges were prepared based on the protocols described in refs. ^80,121^. In brief, we prepared 12.5% (w/w) dispersions by mixing long-chain (DMPC or DMPG) and short-chain (DHPC) phospholipids (Avanti Polar Lipids) in chloroform or chloroform:methanol 65:35, evaporating the solvent under a nitrogen flow and rehydrating the resulting lipid film in 20 mM sodium phosphate, 150 mM NaCl, 1 mM EDTA pH 6.5. The mixtures underwent 35 freeze-thaw cycles with vortexing until the lipid suspension was clear and transparent and then checked by dynamic light scattering. The molar ratio of lipids in bicelle samples was 1.0 DHPC:0.8 DMPG (*q* = 0.8, 44.4% DMPG molar ratio) and 1.0 DHPC:0.8 DMPC (*q* = 0.8, 44.4% DMPC molar ratio). The *q*-ratio (DMPC or DMPG)/DHPC) of 0.8 provides isotropic fast tumbling particles. To investigate C-Tir:bicelle interaction, ^15^N-labelled protein was added to the bicelles to a final concentration of 115 μM protein and 6% (w/v) of lipids. Lipid-binding effects were determined as the ratio of [^15^N-^1^H^N^]-HSQC cross peak intensity of the samples in the absence and presence of bicelles.

### Tir lipid-binding site mutations

The pSA10-Tir_WT_ plasmid was used as a template for mutations. Primer sequences are listed in Supplementary Table 6. R388 and R389 were mutated to glutamic acid using primer Tir­_388E389E_Fwd and Tir­_388E389E_Rvs. L513 were mutated to glutamic acid using primer Tir­_513E_Fwd and Tir­_513E_Rvs. STOP codon was introduced at A541 using primer Tir_541*_Fwd and Tir_541*_Rvs. The resulting plasmids were transformed into *E. coli* DH5α, isolated and sequenced by Eurofins using primer Tir_CT_seq before the next round of mutation. To create pSA10-Tir_388E389E513E_, a second round of mutation was performed using primer Tir­_513E_Fwd and Tir­_513E_Rvs to introduce the 513E mutation into pSA10-Tir_388E389E_. To create pSA10-Tir_388E389E513E541*_, a third round of mutation was performed using primer Tir_541*_Fwd and Tir_541*_Rvs to introduce the 541* STOP codon into pSA10-Tir_388E389E513E_. The resulting plasmids were also transformed into *E. coli* DH5α, isolated and sequenced using primer Tir_CT_seq. All sequencing-verified plasmids (Supplementary Table 7) were electroporated into EPEC-0.

### EPEC infection

SNU-C5 cells were seeded at 110^5^ on glass coverslips in each well of a 24-well plate (immunofluorescence) or 510^4^ in each well of a black clear bottom 96-well plate (PI uptake) in Park Memorial Institute (RPMI) medium (Sigma-Aldrich) with 10% (v/v) foetal bovine serum (FBS) (Gibco), 2 mM Glutamax (Gibco), 1 mM sodium pyruvate, 10 mM HEPES, and 2,5 μg/ml glucose. SNU-C5 cells were primed by IFNγ at 10 µg/ml 24 h before infection. EPEC E2348/69 strains were cultured at 37 °C 180 rpm shaking overnight in Luria Bertani (LB) medium and sub-cultured 1:50 in unsupplemented Dulbecco’s Modified Eagle Medium (DMEM) (low glucose) for 3 h at 37 °C with 5% CO_2_ static before infection. IPTG at 0.5 mM was added to the bacterial culture to induce protein expression from the pSA10 plasmid 30 min before infection. Infection was performed at a multiplicity of infection of 50:1. Infected cells were centrifuged at 700 g for 10 min and incubated at 37 °C with 5% CO_2_ static for required time.

### Immunofluorescence imaging

Infection was performed as described above. At 2 h post-infection, infected cells were fixed by 4% paraformaldehyde for 15 min, washed by PBS for 3 times, permeabilized by 0.2% Triton X-100 in PBS for 4 min, washed again by PBS and blocked by 1% bovine serum albumin (BSA) for 10 min. Samples was incubated with rabbit anti-EPEC serum (VLA) (1:1000) in 1% BSA for 45 min, washed by PBS, re-blocked and incubated with the donkey anti-rabbit IgG-Alexa 488 antibody (Jackson Immunoresearch) (1:200), phalloidin-TRITC (Sigma-Aldrich) (1:200) and DAPI (Sigma-Aldrich) (1:1000) in 1% BSA for another 30 min and washed by PBS afterwards. Coverslips were mounted on glass slides (VWR) with Gold-Pro-Long-Anti-fade (Invitrogen). Imaging was performed using a Zeiss AxioImager Z1 microscope (Carl Zeiss) equipped with 100 lens.

### Propidium iodide uptake assay

Infection was performed as described above. Cells were incubated with 5 µg/ml propidium iodide (PI) (Invitrogen) from 1 h before infection in phenol red-free serum-free RPMI medium. Positive control was performed by treating uninfected cells incubated in 0.05% Triton X-100 in PI-supplemented RPMI medium. Uninfected cells were used as negative control. No cell medium-only wells were used as blank. Time-course measurements were carried out in a FLUOstar Omega Microplate Reader (BMG Labtech) from 10 min to 8 h post-infection with 10-min intervals, measuring 620-nm emission with 520-nm excitation. After 2 h post-infection, 200 µg/ml gentamicin (Sigma-Aldrich) was added to each well. All PI uptake data were blank-corrected and normalised to negative control. Increase in percentage PI uptake was then calculated using the normalised PI uptake value of the infected sample divided by the normalised PI uptake value of the positive control. Statistical analysis of PI uptake assay was performed using GraphPad Prism 5.1.

### Statistics and reproducibility

We compared disorder fraction distributions with the Mann-Whitney U-test. All biochemical experiments were performed at least three times. SAXS experiments were performed in duplicates at independent facilities (ESRF-BM29 and DLS-B21 Bio-SAXS beamlines) using different protein samples. All NMR samples were freshly prepared, followed by immediate recording of NMR spectra with multiple scans to ensure a good signal-to-noise ratio. NMR spectra were reproduced on different samples and also using technical replicates. Cell-based assays were performed with at least 3 biological replicates. Statistical analysis was performed using 1-way ANOVA with Tukey’s post-test. *p ≤ 0.05; **p ≤ 0.01; ***p ≤ 0.001; ns: non-significant.

### Reporting summary

Further information on research design is available in the Nature Portfolio Reporting Summary linked to this article.

### Supplementary information

Supplementary Information

Description of Supplementary Materials

Supplementary Data 1

Reporting Summary

## Data Availability

The NMR chemical shifts of C-Tir, pC-Tir and NS-Tir are available at the BMRB with accession codes 50758 (C-Tir), 50759 (pC-Tir) and 52064 (NS-Tir). The SEC-SAXS data and models are available at SASBDB)^122^ under the project “SAXS studies on the intracellular region of the translocated intimin receptor”. The accession codes are detailed in Supplementary Table 5. Proteomes and effector collections, disorder predictions in Fig. 1 are available for download at https://osf.io/3mka9/ and supplementary material. A PRE/SAXS-based structural sub-ensemble of 200 structures for C-Tir is in the open-access Protein Ensemble Database (PED)^123^ with the identifier code PED00210. Numerical source data for graphs in Figs. 2 to 9 can be found in the supplementary data 1 file.

## Code availability

The associated code is available at https://osf.io/cxkjf/.
