# Supplementary material for: The pathogen-encoded signalling receptor Tir exploits host-like intrinsic disorder for infection
Source: Commun Biol. 2024 Feb 13;7:179. doi: 10.1038/s42003-024-05856-9 (PMC10864410; doi:10.1038/s42003-024-05856-9)
Supplement: Supplementary file 1 — Supplementary Information [file 42003_2024_5856_MOESM1_ESM.pdf]

# Supplementary Information

# The pathogen-encoded signalling receptor Tir exploits host-like intrinsic disorder for infection

Marta F. M. Vieira[1†], Guillem Hernandez[1†], Qiyun Zhong[2], Miguel Arbesú[3#], Tiago Veloso[1], Tiago Gomes[1], Maria L. Martins[1], Hugo Monteiro[1], Carlos Frazão[1], Gad Frankel[2], Andreas Zanzoni[4], Tiago N. Cordeiro[1,*]

[1] Instituto de Tecnologia Química e Biológica António Xavier, Universidade Nova de Lisboa, Av. da República, 2780-157 Oeiras, Portugal
[2] Department of Life Sciences, Imperial College London, South Kensington Campus, London SW7 2AZ, UK
[3] Department of NMR-supported Structural Biology, Leibniz-Forschungsinstitut für Molekulare Pharmakologie, Robert-Rössle-straße 10, 13125 Berlin, Germany
[4] Aix-Marseille Université, Inserm, TAGC, UMR_S1090, Marseille,France

* To whom correspondence should be addressed. Email: tiago.cordeiro@itqb.unl.pt

† These authors contributed equally to this work

# Present address: InstaDeep Ltd, 5 Merchant Square, London, W2 1AY, UK

## Supplementary Information Statement

Figures showing predicted structural disorder in A/E pathogens with IUPred (Supplementary Figure 1); A/E disordered effectors (Supplementary Figure 2); Tir's sequence alignment conservation (Supplementary Figure 3); SEC and SDS-page of Tir constructs (supplementary Figure 4); SEC-SAXS chromatograms of Tir constructs (Supplementary Figure 5); SAXS profiles of Tir variants compared against globular proteins with similar molecular mass (Supplementary Figure 6); Order and disorder in N-Tir (Supplementary Figure 7); Thermal denaturation of NS-Tir monitored by far-UV CD spectroscopy (Supplementary Figure 8);) Reverse labelling of C-Tir (Supplementary Figure 9); $^{1}H$-$^{15}N$ PRE profiles of C-Tir (Supplementary Figure 10); AF-2 predicted C-Tir:C-SH2 complex (Supplementary Figure 11); Tyrosine phosphorylation of C-Tir (Supplementary Figure 12); NMR quantification of the binding of C-SH2 to individual pY-sites (Supplementary Figure 13); Impact of TFE on C-Tir (Supplementary Figure 14); Time course PI uptake of EPEC-infected cells (Supplementary Figure 15); Reference proteomes by species name and taxon (Supplementary Table 1); Structural categories by disorder content (Supplementary Table 2); Disorder fractions, predicted motif densities, and structural categories for A/E effectors (Supplementary Table 3); Protein constructs expressed and purified in this study (Supplementary Table 4); SAXS data collection and analysis (Supplementary Table 5); Primers information (Supplementary Table 6); Plasmids list (Supplementary Table 7).

## Supplementary Figures

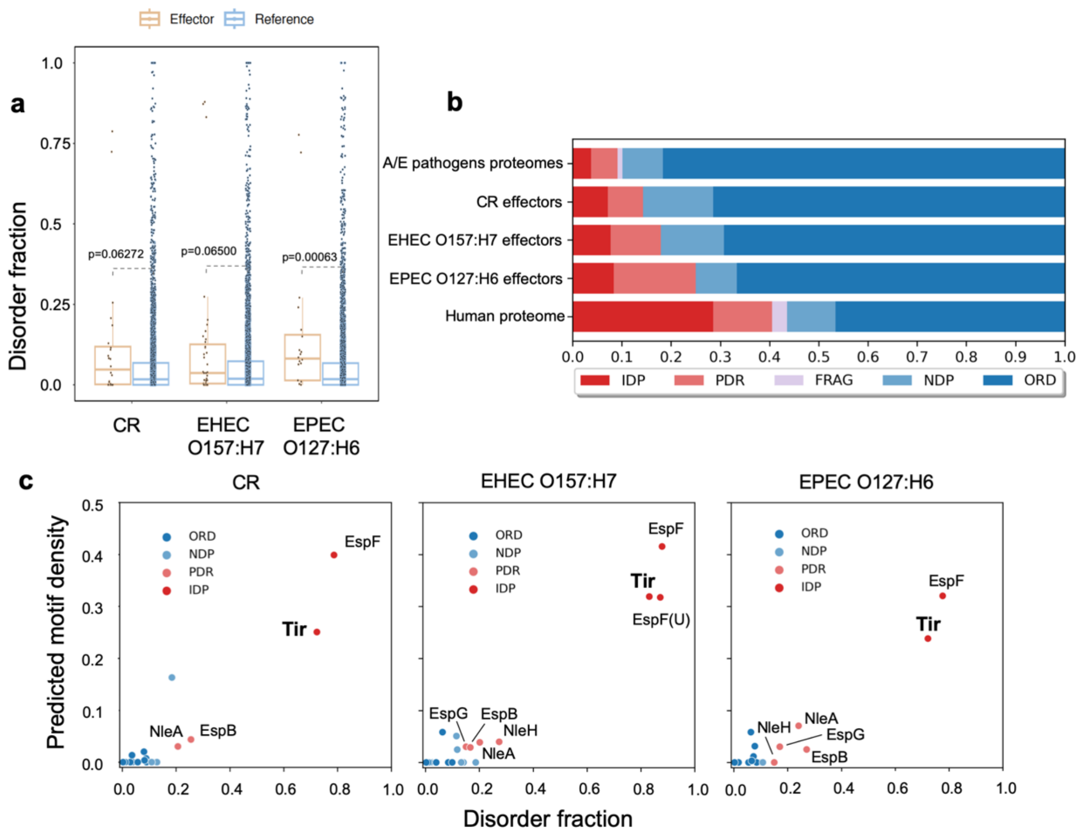


**Supplementary Figure 1. Predicted structural disorder in A/E pathogens with IUPred. a** Distribution of IUPred-based disorder fraction displayed as violin plots of effectors (orange) and full-proteomes (blue) of A/E pathogens. Statistical analysis with Mann Whitney U-test. **b** Accumulated fractions of the structural categories in terms of sequence disorder based on IUPred disorder-score (*long* mode) as described for **Figure 1**. **c** Fraction of ELMs vs. disorder fraction in A/E effectors. Tir, EspF, and EspF(U) display a high motif content and disorder fraction. IDP-like (red dots) and PDR (orange) effectors are labelled.

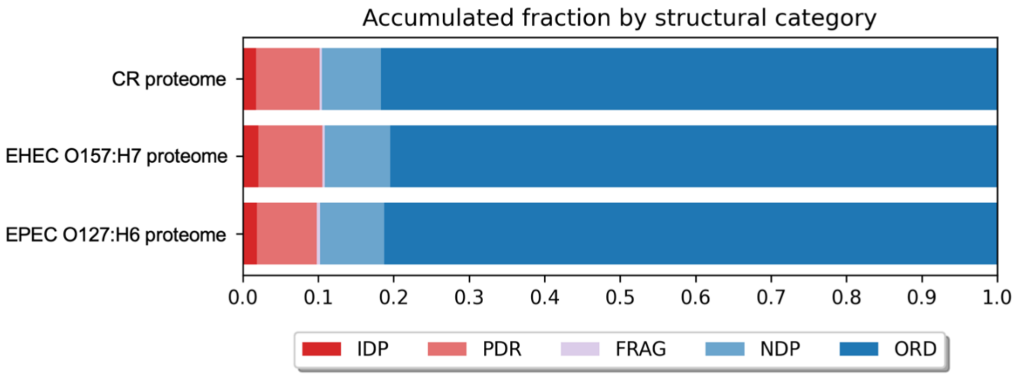


**Supplementary Figure 2. Order/disorder propensity of individual A/E pathogen proteomes.** Accumulated fractions of structural categories based on sequence disorder for the individual A/E pathogen reference proteomes (see **Figure 1** and **Supplementary Table 1**).

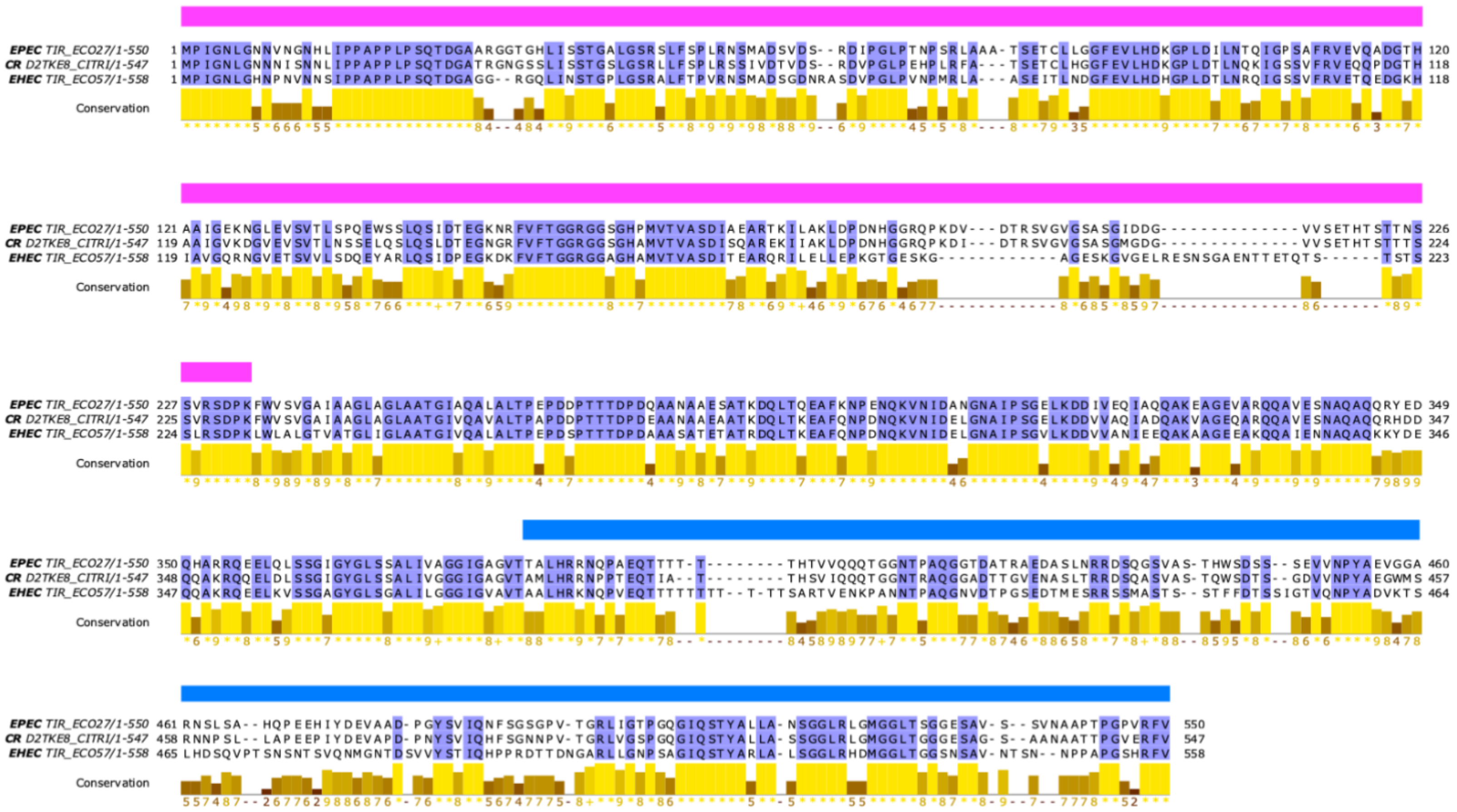

**Supplementary Figure 3. Tir's sequence alignment conservation.** Multiple sequence alignment (MSA) from a set of 114 non-identical sequences belonging to the Tir receptor family from the Uniprot database using the CD-HIT algorithm[1]. The MSA was generated using the Clustal Omega program[2] with default parameters, and we used JalView [3] for MSA visualisation and figure generation. For convenience, only Tir sequences from EPEC O127:H6, CR strain ICC168, and EHEC O157:H7 are shown. Alignment conserved positions are shaded in lavender. The conservation of physicochemical properties in each alignment position is reported in the corresponding barplot below the alignment. The intracellular domains of EPEC Tir are delimited by magenta (N-Tir) and light-blue (C-Tir) horizontal boxes, respectively.

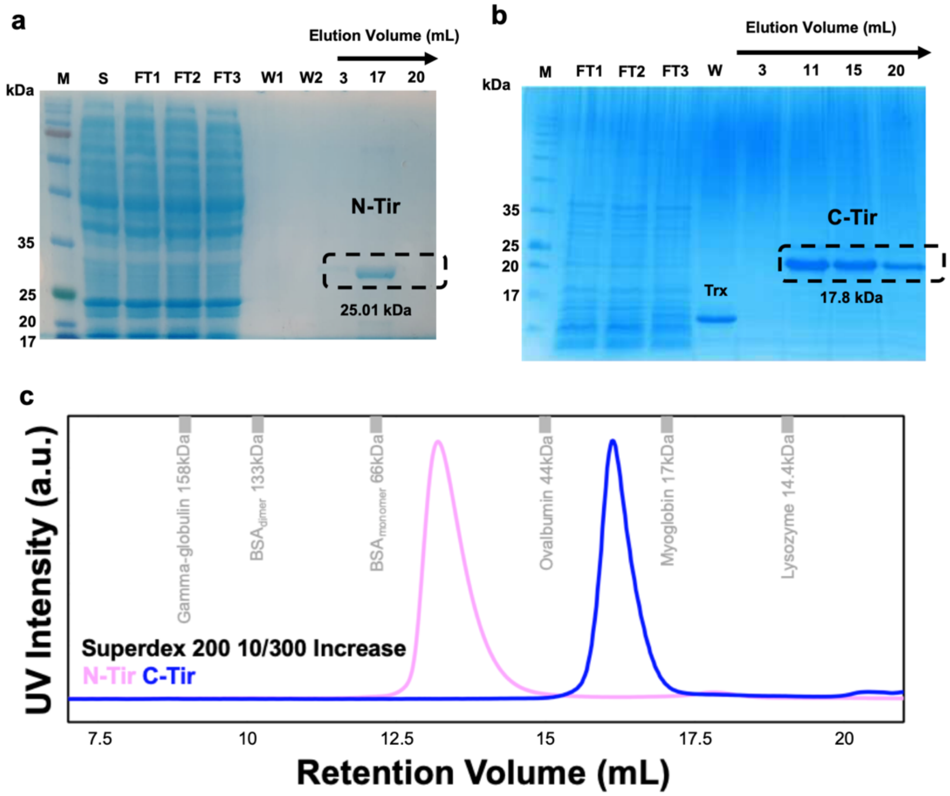


**Supplementary Figure 4. Expression and purification of Tir's intracellular domains** N-Tir and C-Tir were expressed as fusion proteins with a cleavable N-terminal thioredoxin tag (Trx-His6) and C-terminal Strep-tag. **a, b** Reducing SDS-PAGE analysis of N-Tir and C-Tir purification from *E.coli* BL21 Star (DE3) pLysS cleared lysate (lane S) loaded onto a Strep-Tactin column. Lanes M are protein size standards. Lanes FT1, 2, and 3 correspond to column flow-through fractions. **a** The column was washed twice with 5ml of washing buffer before elution (lanes W, W1, W2). **b** Following on-column cleavage, Trx-His6 was washed from the column before elution. Both constructs elute as a single band with no signs of protein degradation. **c** Size exclusion chromatograms for C-Tir (blue) and N-Tir (pink) eluting from a Superdex 200 10/300 increase gel filtration column. For reference, the retention volume of markers is indicated by grey squares.

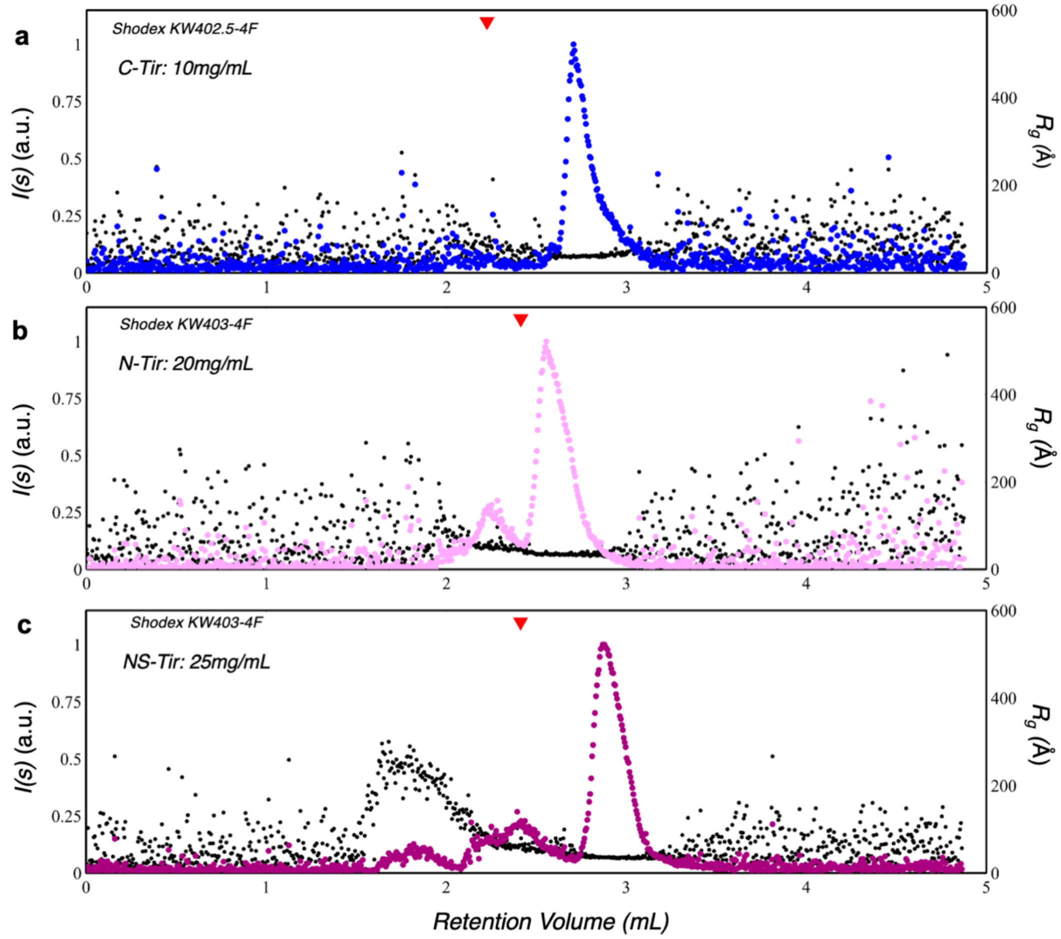


**Supplementary Figure 5.** SEC-SAXS chromatograms of **a** C-Tir, **b** N-Tir, and **c** NS-Tir. Each plane shows normalised total scattering intensity *I(s)*, over the entire s range, from each frame acquired along elution volume and respective $R_g$-value (black circles). The flat variation of $R_g$ values reflects a pure monodisperse sample. The column type for high-resolution size exclusion chromatography and sample concentrations are on the top left of each panel. For reference, the retention volume for monomeric BSA (66.4 kDa) is displayed by red triangles.

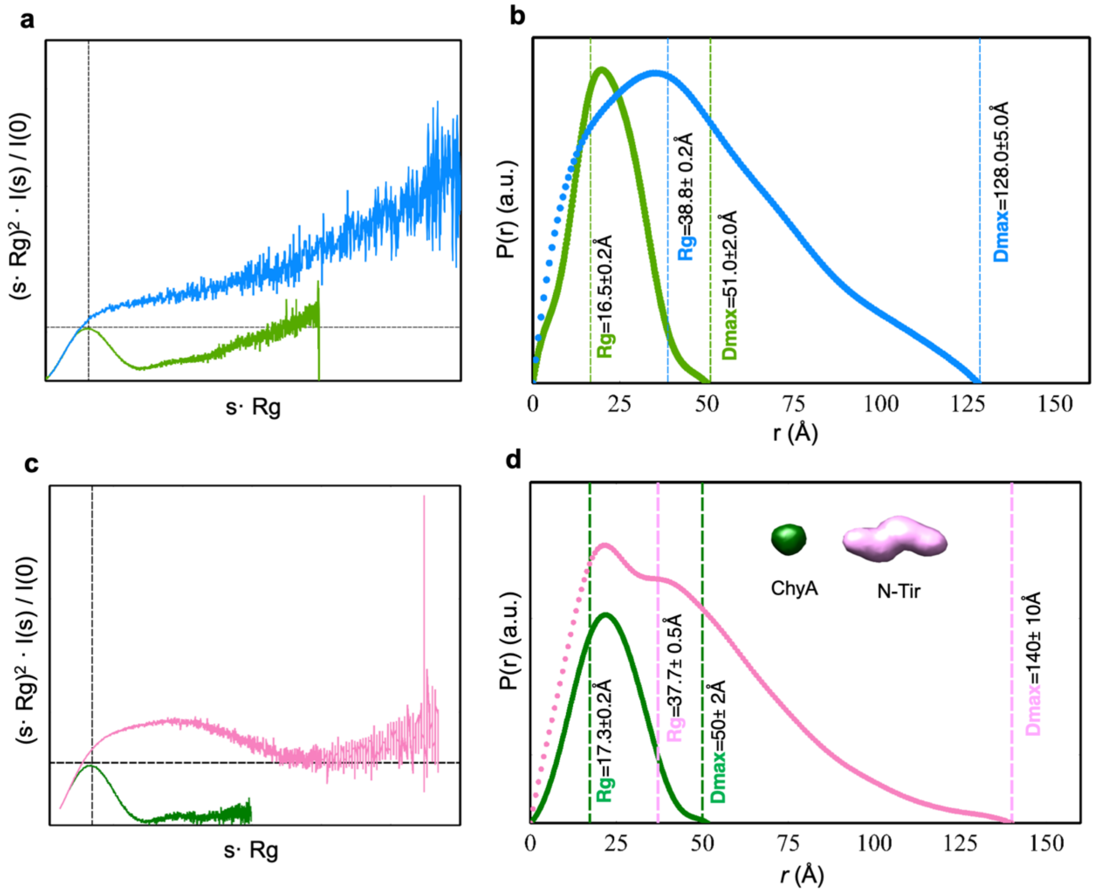


**Supplementary Figure 6. Comparative SAXS: a, b C-Tir vs globular Myoglobin and c, d N-Tir dimer vs Chymotrypsinogen A.** Myoglobin (Myb) and Chymotrypsinogen A (ChyA) are monomeric globular proteins with a molecular mass similar to Strep-tagged C-Tir (ca 17 kDa) and N-Tir monomer (ca 26 kDa), respectively. **a** Kratky representations of the SAXS patterns of C-Tir (blue) and Myb (green) are compared next to their respective *P(r)* versus *r* profiles and plotted using the same color code. The absence of a clear peak maximum at $sRg = \sqrt{3}$ (dashed cross) indicates that C-Tir is conformationally flexible and non-globular. In contrast, the Kratky plot of Myb is bell-shaped as expected for well-folded globular proteins. The SAXS experimental set for Myb was obtained from the curated repository of scattering data SASDB (www.sasbdb.org) [5], with the entry code SASDAH2. **b** Normalized pairwise distance distribution, P(r), computed from experimental SAXS curves of C-Tir (blue) and Myb (green). Dashed lines indicate the derived *Rg* and *Dmax* values. C-Tir's internal distances are more significant than in Myb, as expected for a more extended and non-globular protein. **c** Kratky representation of the SAXS patterns of N-Tir (pink) and ChyA (green). Also, for N-Tir, the absence of a peak maximum at $sRg = \sqrt{3}$ (dashed line) indicates that N-Tir is highly aspherical. **d** Respective *P(r)* versus r profiles plotted using the same color code with derived *Rg* and *Dmax* values indicated by dashed lines. N-Tir dimer *P(r)* displays two prominent peaks that encapsulate the intra- and interdomain pairwise distances of N-Tir protomers, respectively. The high-density core of the low-resolution reconstruction of N-Tir (pink; DAMFILT-model reveals a non-globular particle, contrasting with the model of ChyA obtained using the SAXS data from SASBD with the entry code SASDAA8.

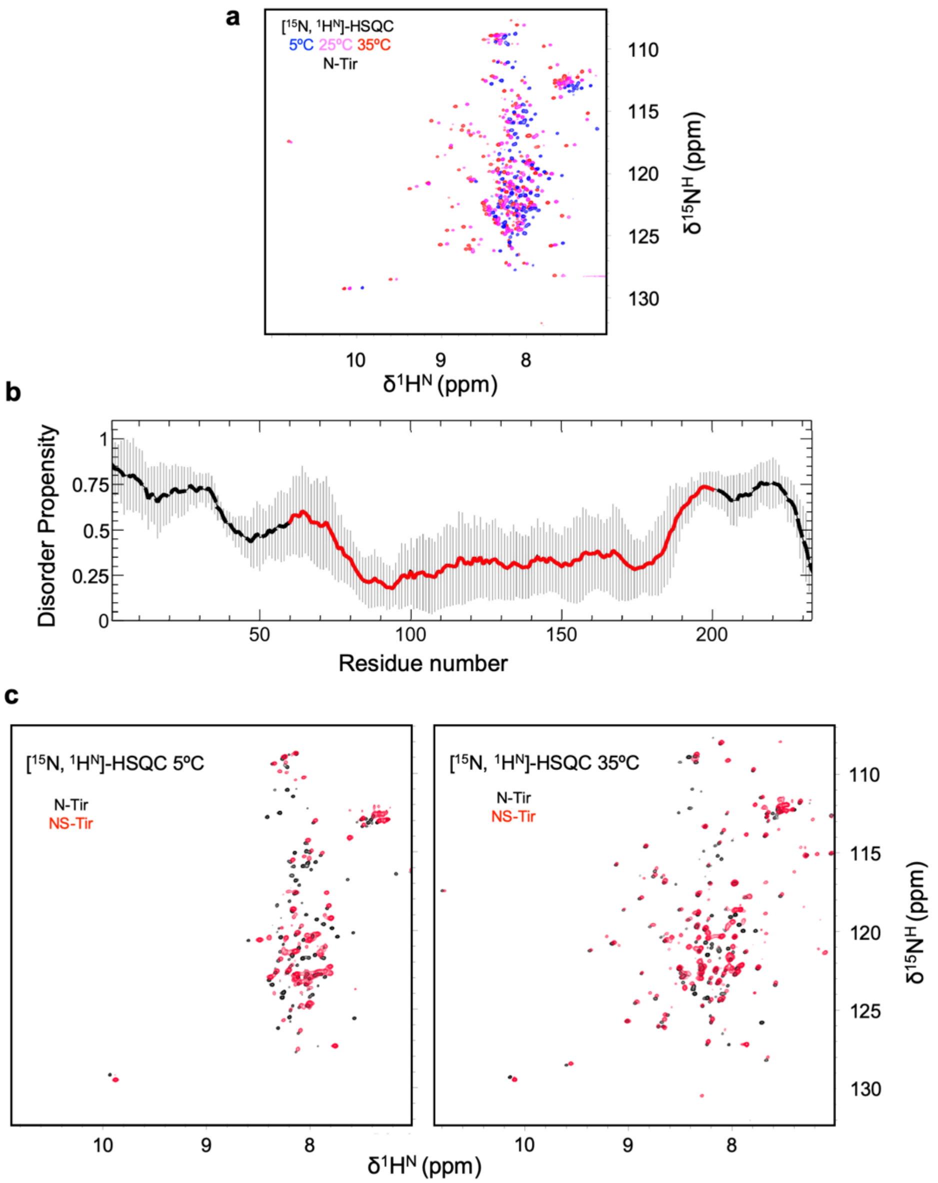


**Supplementary Figure 7. Order and disorder in N-Tir. a** [$^{15}$N, $^{1}$H$^{N}$]-HSQC NMR spectra of N-Tir at 5, 25 and 35ºC. **b** Average disorder prediction and its standard deviation (error bars) computed using four different computational tools along the primary sequence of N-Tir. Contrary to NS-Tir (i.e., N-Tir$_{60\text{-}200}$; solid red), the flanking regions (black dashed-lines) have a clear disorder tendency, as consensually assessed with IUPRED[6], PrDOS [7], PONDR-FIT[8], and DISOPRED3[9]. **c** Overlay of [$^{15}$N, $^{1}$H$^{N}$]-HSQC NMR spectra of N-Tir (black) and NS-Tir (red) at 5 ºC (left panel) and 35 ºC (right panel).

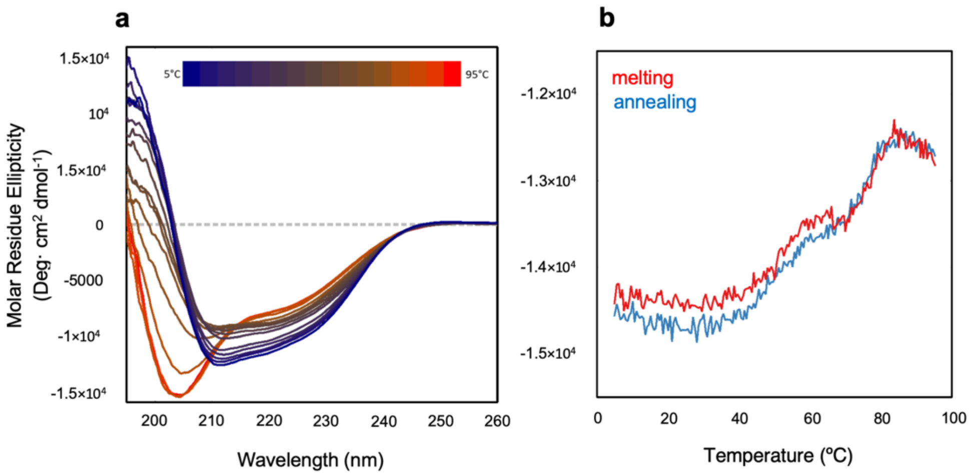


**Supplementary Figure 8 - Thermal denaturation of NS-Tir monitored by far-UV CD spectroscopy. a** Far-UV CD spectra of NS-Tir recorded from 5 ºC to 95 ºC, revealing the loss of structure. **b** CD signal changes at 222 nm during the temperature ramp, showing a two-step transition.

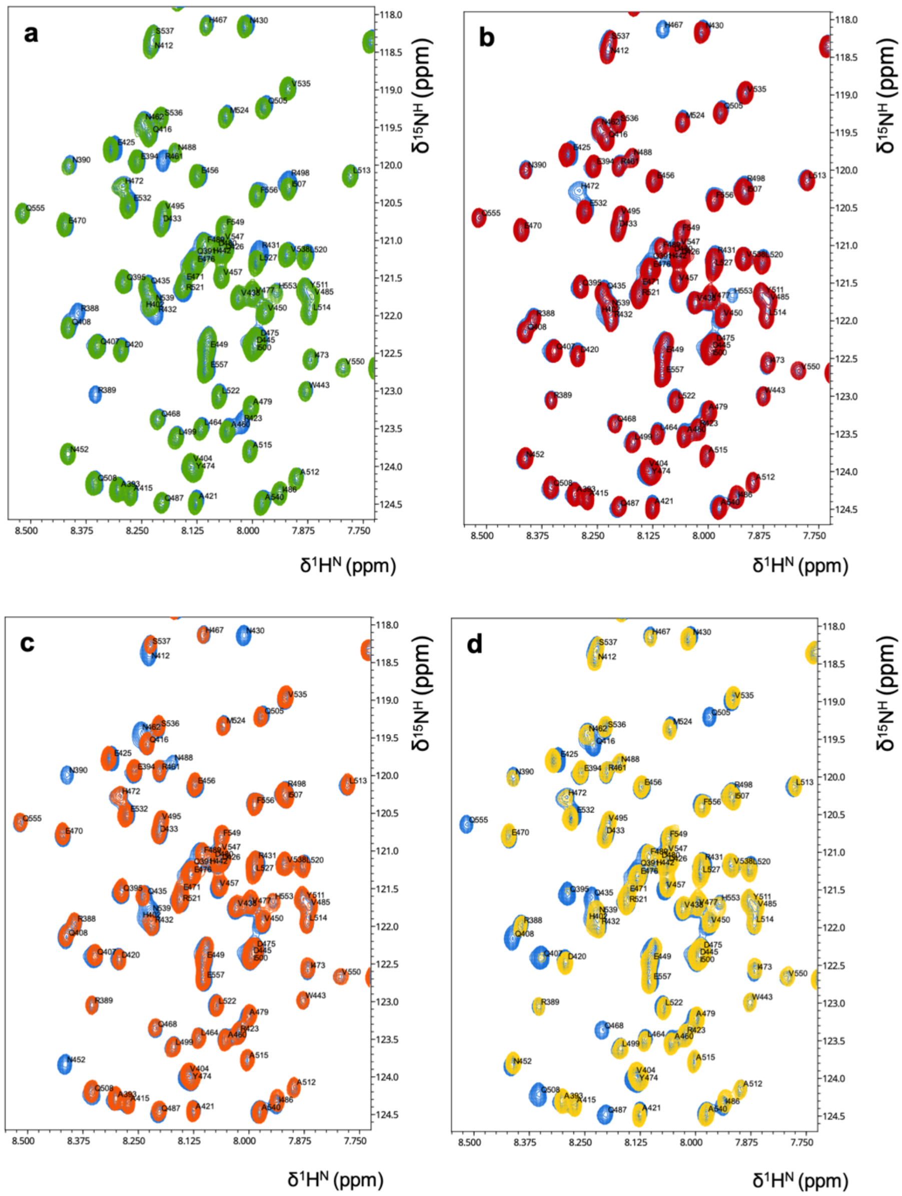


**Supplementary Figure 9. Reverse labelling of C-Tir.** The [$^{15}$N-1H$^{N}$]-HSQC NMR spectrum of C-Tir exhibits a characteristic spectral crowding in the random-coil region around 8.0 ppm. To alleviate the ambiguity caused by substantial resonances overlapping, we selectively unlabeled **a** Asparagine, **b** Histidine, **c** Lysine, and **d** Glutamine residues against a uniformly $^{15}$N-labelled C-Tir (blue). Selectively unlabeled residues became absent in the HSQC spectra due to reverse labelling, allowing pinpointing of the amino-acid type. We used this prior knowledge to aid and validate the assignment.

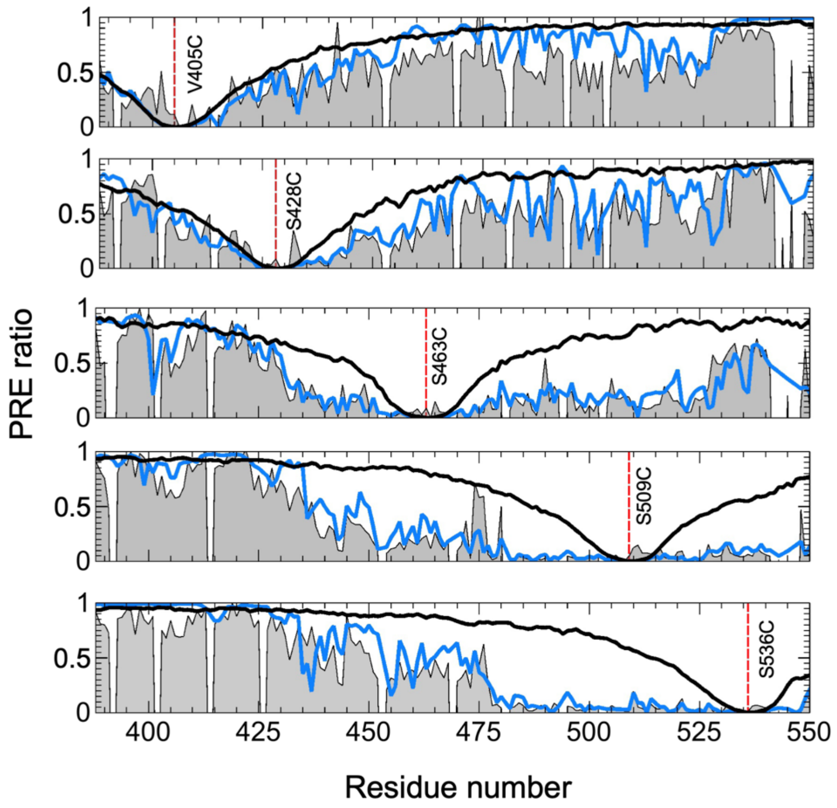


**Supplementary Figure 10. $^1H$-$^{15}N$ PRE profiles.** PRE data (grey) from five single cysteine C-Tir mutants with an MTSL at position V405C, S428C, S463C, S509C, and S536C top to bottom, respectively. Back-calculated PRE ratios from an ideal random coil ensemble (solid black lines) and from a set of 200 conformers selected using EOM (solid blue line).

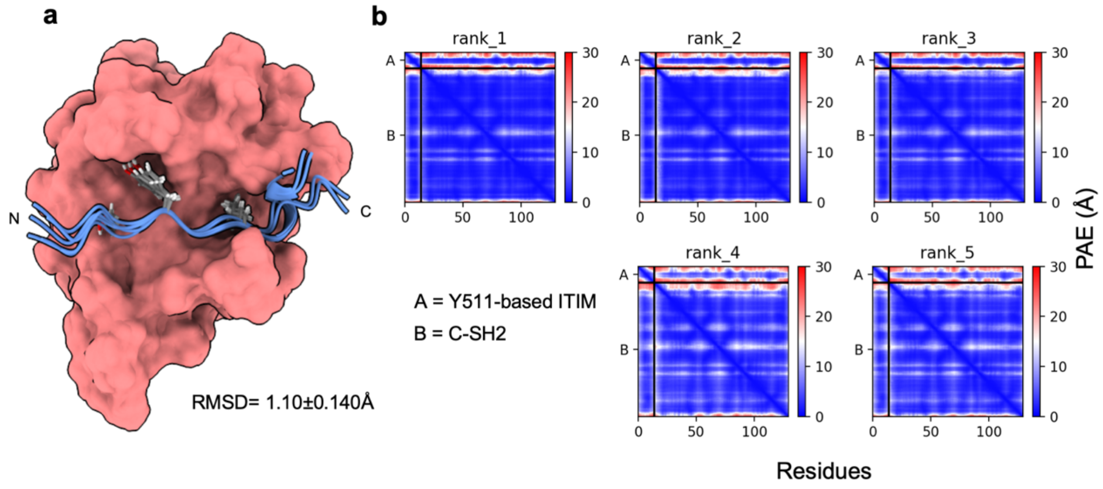


**Supplementary Figure 11 - C-Tir:C-SH2 predicted complexes. a** Ribbon representation of the five AF2-based models with unphosphorylated C-Tir (light blue) bound to C-CH2 (pink), with an average RMSD of 1.10±0.14Å from the top-ranked model. The interface residues are in stick format. **b** Predicted Aligned Errors (PAE) on a 0 to 30 Å scale in a blue-white-red gradient. PAE is a metric of confidence in the relative position and orientation of the different chains of the model (i.e. A=C-Tir Y511 ITIM, B=C-SH2). All models display low inter-chain PAE values (blue), indicative of well-defined relative positions and orientations within the predicted complexes.

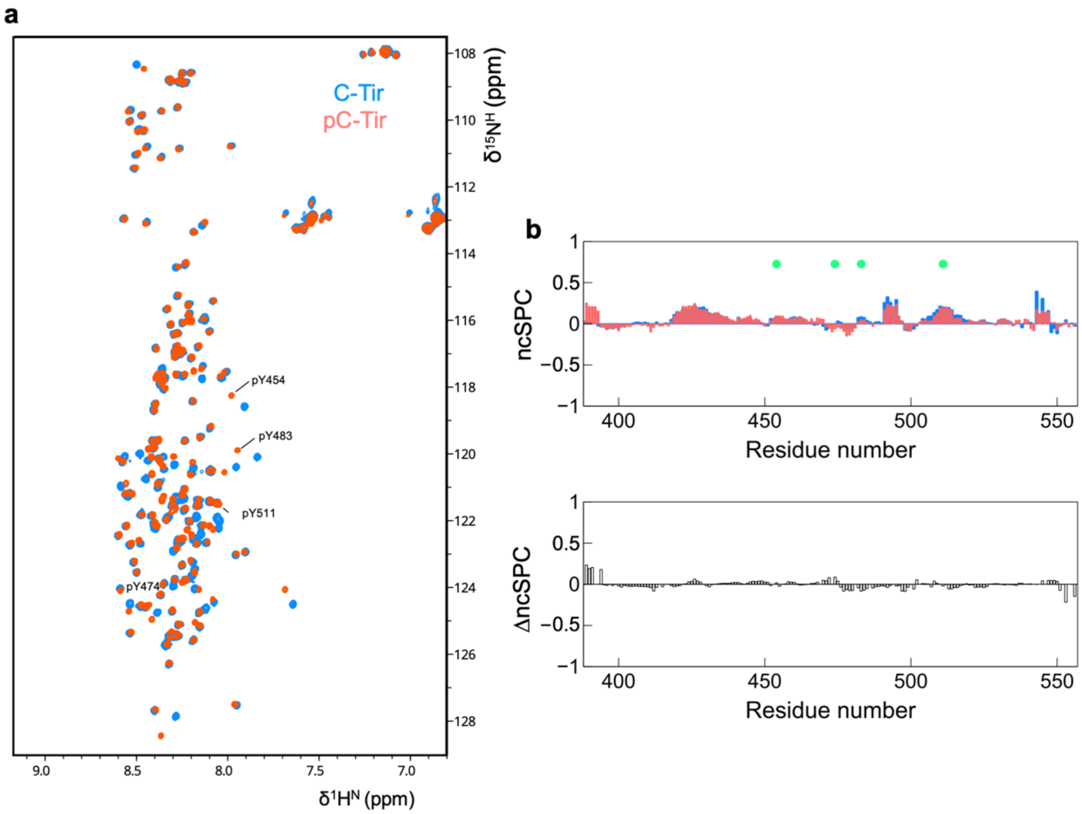


**Supplementary Figure 12. Tyrosine phosphorylation of C-Tir. a** Overlay of [$^{1}$H-$^{15}$N]-HSQC NMR spectra of C-Tir (blue) and pC-Tir (orange) tyrosine-phosphorylated overnight. In the pC-Tir sample, resonances of residues pY545, pY474, pY483, pY511 exhibit proton downfield shifts accompanied by small chemical shift changes in adjacent residues. **b** Neighbour-corrected sequence structural propensity score (top) of C-Tir (blue) and pC-Tir (orange). Minor differences in ncSCP were observed upon phosphorylation (bottom).

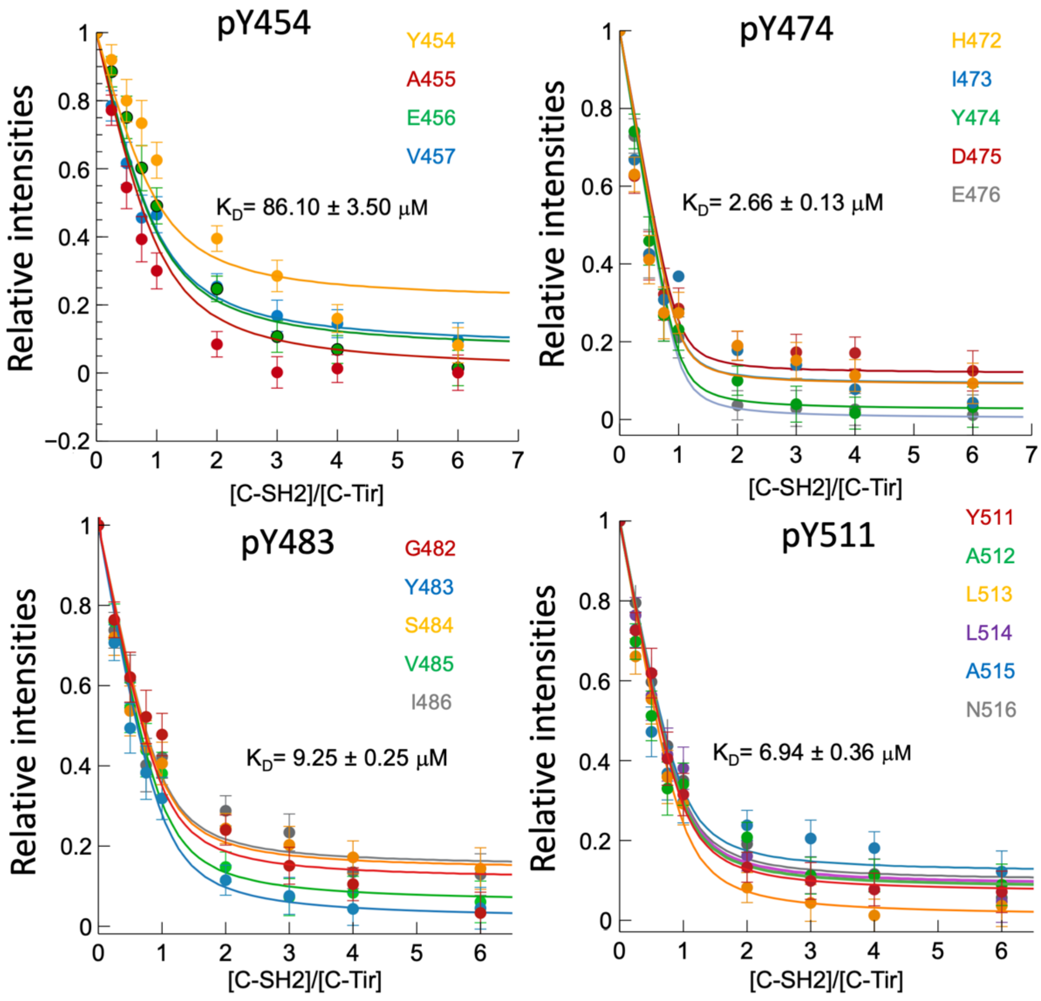


**Supplementary Figure 13. NMR quantification of the binding of C-SH2 to individual pY-sites.** Relative apparent $K_D$ values obtained by globally fitting a 1:1 model (eq. 1) to the relative local intensity drop around each phosphorylation site (i.e., each phospho-tyrosine (pY) and neighbouring residues). The data shown are normalized intensities by the initial peak height without C-SH2. The error bars are the uncertainty (ΔR) from random noise in the ratio of two peak heights, i.e., $\Delta R = (\Delta A.B - A.\Delta B)/B^2 = \Delta A/B - A^*\Delta B/B^2$, where ΔA and ΔB are the uncertainties in the peak heights. The solid lines correspond to the fitting.

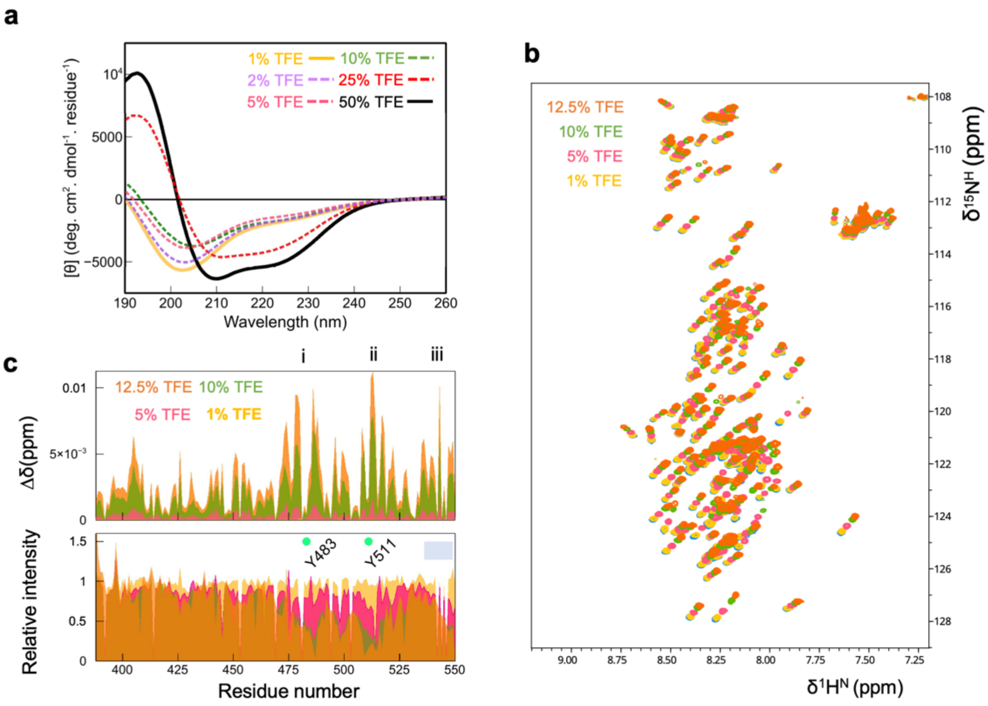


**Supplementary Figure 14. Impact of TFE on C-Tir. a**) Far-UV CD and **b**) NMR [$^{15}$N-1H$^{N}$]-HSQC spectra of C-Tir in the presence of increasing amounts of TFE, showing C-Tir gradually acquiring structure. Most cross-peaks in the $^{15}$N-1H$^{N}$ plane move upfield in both dimensions, even after referencing all chemical shifts to DSS. **c**) Residue-resolution mapping of the effect of TFE on C-Tir. TFE-induced chemical shift perturbations (Δδ, top) as a function of residue number, with 3-regions displaying Δδ higher than the mean plus one standard deviation, including Y483, Y511, and C-Tir C-terminal residues, respectively. TFE-induced NMR attenuation profiles (bottom), i.e., peak intensity ratio in the presence and absence of TFE, are plotted along the sequence. Green circles mark the position of Y483 and Y511. The grey bar highlights the C-terminal residues with NMR-signal attenuation.

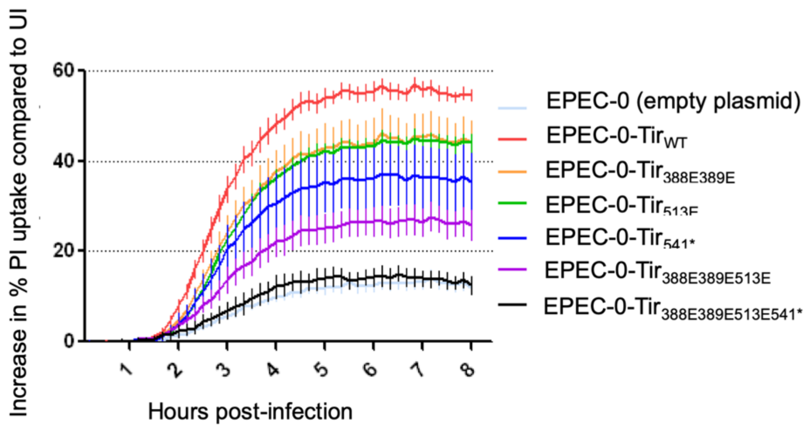


**Supplementary Figure 15. Time course PI uptake of EPEC-infected cells.** PI uptake into SNU-C5 cells infected with EPEC-0 (empty plasmid), EPEC-0-Tir$_{WT}$, EPEC-0-Tir$_{388E389E}$, EPEC-0-Tir$_{513E}$, EPEC-0-Tir$_{541*}$, EPEC-0-Tir$_{388E389E513E}$ or EPEC-0-Tir$_{388E389E513E541*}$ from 10 min to 8 h post-infection with 10 min intervals. Data shown are meanSEM from 3 (EPEC-0-Tir$_{388E389E513E541*}$) or 6 (all other strains) independent biological repeats.

## Supplementary Tables

**Supplementary Table 1.** Reference proteomes by species name and taxon.

| Specie | A/E type | Taxon | Reference Uniprot proteome |
|---|---|---|---|
| *Homo sapiens* | - | 9606 | UP000005640 (Swiss-Prot reviewed) |
| *E. coli* O157:H7 | EHEC | 83334 | UP000000558 (Full) |
| *E. coli* O127:H6 | EPEC | 574521 | UP000008205 (Full) |
| *C. rodentium* (strain ICC168) | CR | 637910 | UP000001889 (Full) |

**Supplementary Table 2.** Structural categories by disorder content adapted from [10]

| Class | Description | Average disorder content | ≥ 22 consecutive disordered residues |
|---|---|---|---|
| IDP | Intrinsically disordered proteins | ≥ 30% | Yes |
| PDR | Proteins with intrinsically disordered regions | < 30 % | Yes |
| FRAG | Proteins with fragmented disorder | ≥ 30 % | No |
| NDR | Not disordered proteins | 10 % ≤ x < 30 % | No |
| ORD | Ordered Proteins | < 10 % | No |

**Supplementary Table 3.** Disorder fractions (DISOPRED), predicted motif densities and structural categories for CR (strain ICC168) , EHEC O157:H7 and EPEC O127:H6 effectors in **Fig. 1C.**

| protein_ac | disorder_fraction | overall_motif_density | disorder_category | effector_collection |
|---|---|---|---|---|
| A0A0H3JC80 | 0.007 | 0.000 | ORD | EHEC |
| A0A0H3JD33 | 0.073 | 0.000 | ORD | EHEC |
| A0A0H3JDH6 | 0.011 | 0.000 | ORD | EHEC |
| A0A0H3JDV8 | 0.032 | 0.000 | ORD | EHEC |
| A0A0H3JE38 | 0.091 | 0.000 | ORD | EHEC |
| A0A0H3JFN8 | 0.077 | 0.000 | ORD | EHEC |
| A0A0H3JGR6 | 0.095 | 0.000 | ORD | EHEC |
| A0A0H3JHA4 | 0.163 | 0.000 | NDP | EHEC |
| A0A0H3JJJ4 | 0.007 | 0.000 | ORD | EHEC |
| A0A0H3JP21 | 0.020 | 0.000 | ORD | EHEC |
| P0DJ88 | 0.424 | 0.318 | IDP | EHEC |
| Q7DB50 | 0.048 | 0.030 | ORD | EHEC |
| Q7DB68 | 0.051 | 0.000 | ORD | EHEC |
| Q7DB74 | 0.125 | 0.000 | NDP | EHEC |
| Q7DB76 | 0.148 | 0.025 | PDR | EHEC |
| Q7DB77 | 0.332 | 0.319 | IDP | EHEC |
| Q7DB85 | 0.601 | 0.415 | IDP | EHEC |
| Q7DBA6 | 0.045 | 0.000 | ORD | EHEC |
| Q8X4Q6 | 0.061 | 0.000 | ORD | EHEC |
| Q8X4W3 | 0.077 | 0.000 | ORD | EHEC |
| Q8X4X1 | 0.068 | 0.000 | ORD | EHEC |
| Q8X4X2 | 0.072 | 0.000 | ORD | EHEC |
| Q8X4X3 | 0.094 | 0.000 | ORD | EHEC |
| Q8X507 | 0.089 | 0.000 | ORD | EHEC |
| Q8X509 | 0.068 | 0.000 | ORD | EHEC |
| Q8X782 | 0.075 | 0.000 | ORD | EHEC |
| Q8X831 | 0.201 | 0.000 | PDR | EHEC |
| Q8X834 | 0.052 | 0.000 | ORD | EHEC |
| Q8X9A5 | 0.014 | 0.000 | ORD | EHEC |

| | | | | |
|---|---|---|---|---|
| Q8X9A7 | 0.093 | 0.000 | ORD | EHEC |
| Q8XA11 | 0.056 | 0.000 | ORD | EHEC |
| Q8XAJ5 | 0.068 | 0.039 | ORD | EHEC |
| Q8XAL6 | 0.040 | 0.040 | ORD | EHEC |
| Q8XAL7 | 0.042 | 0.058 | ORD | EHEC |
| Q8XAN6 | 0.093 | 0.000 | ORD | EHEC |
| Q8XB17 | 0.041 | 0.000 | ORD | EHEC |
| Q8XB62 | 0.046 | 0.051 | ORD | EHEC |
| Q8XBX8 | 0.073 | 0.000 | ORD | EHEC |
| Q8XC86 | 0.228 | 0.029 | PDR | EHEC |
| A0A1A9NR20 | 0.074 | 0.000 | ORD | EPEC |
| A0A1E5MD86 | 0.121 | 0.000 | NDP | EPEC |
| B7UH72 | 0.074 | 0.000 | ORD | EPEC |
| B7UI20 | 0.027 | 0.000 | ORD | EPEC |
| B7UI21 | 0.073 | 0.000 | ORD | EPEC |
| B7UI22 | 0.045 | 0.000 | ORD | EPEC |
| B7UI23 | 0.285 | 0.011 | PDR | EPEC |
| B7ULW4 | 0.147 | 0.000 | PDR | EPEC |
| B7ULW8 | 0.083 | 0.000 | ORD | EPEC |
| B7UM88 | 0.592 | 0.320 | IDP | EPEC |
| B7UM99 | 0.400 | 0.238 | IDP | EPEC |
| B7UMA0 | 0.143 | 0.000 | PDR | EPEC |
| B7UMA2 | 0.113 | 0.000 | NDP | EPEC |
| B7UMA9 | 0.051 | 0.000 | ORD | EPEC |
| B7UMC8 | 0.048 | 0.030 | ORD | EPEC |
| B7UNX2 | 0.068 | 0.031 | ORD | EPEC |
| B7UNX4 | 0.052 | 0.000 | ORD | EPEC |
| B7UNX6 | 0.103 | 0.000 | PDR | EPEC |
| B7UR60 | 0.068 | 0.070 | ORD | EPEC |
| B7UR63 | 0.042 | 0.058 | ORD | EPEC |
| Q05129 | 0.277 | 0.025 | PDR | EPEC |
| Q7WRZ5 | 0.110 | 0.000 | PDR | EPEC |

| | | | | |
|---|---|---|---|---|
| Q8VLH6 | 0.073 | 0.000 | ORD | EPEC |
| Q9EZE7 | 0.057 | 0.003 | ORD | EPEC |
| A0A2X2U9X2 | 0.074 | 0.007 | ORD | CR |
| A0A482PJC5 | 0.033 | 0.000 | ORD | CR |
| D2TI12 | 0.076 | 0.163 | ORD | CR |
| D2TI20 | 0.136 | 0.000 | NDP | CR |
| D2TI21 | 0.119 | 0.000 | NDP | CR |
| D2TI55 | 0.011 | 0.000 | ORD | CR |
| D2TJZ4 | 0.074 | 0.030 | ORD | CR |
| D2TK70 | 0.058 | 0.000 | ORD | CR |
| D2TK72 | 0.098 | 0.000 | ORD | CR |
| D2TKD5 | 0.043 | 0.020 | ORD | CR |
| D2TKD7 | 0.877 | 0.399 | IDP | CR |
| D2TKE1 | 0.268 | 0.044 | PDR | CR |
| D2TKE8 | 0.470 | 0.250 | IDP | CR |
| D2TKF1 | 0.078 | 0.000 | ORD | CR |
| D2TKF8 | 0.050 | 0.000 | ORD | CR |
| D2TM85 | 0.094 | 0.000 | ORD | CR |
| D2TML3 | 0.102 | 0.000 | PDR | CR |
| D2TQZ7 | 0.092 | 0.004 | ORD | CR |
| D2TRX7 | 0.065 | 0.000 | ORD | CR |
| D2TRX8 | 0.042 | 0.000 | ORD | CR |
| D2TRY0 | 0.057 | 0.000 | ORD | CR |
| D2TRY1 | 0.088 | 0.000 | ORD | CR |
| D2TT36 | 0.027 | 0.000 | ORD | CR |
| D2TT37 | 0.079 | 0.000 | ORD | CR |
| D2TT38 | 0.083 | 0.000 | ORD | CR |
| D2TTX8 | 0.079 | 0.000 | ORD | CR |
| Q5DKN7 | 0.243 | 0.013 | PDR | CR |
| Q5XMK8 | 0.070 | 0.000 | ORD | CR |

**Supplementary Table 4.** Protein constructs expressed and purified in this study.

| **Construct name** | **Protein sequence**[a] |
|---|---|
| Pathogen-encoded effector | |
| **N-Tir** | *G***1**MPIGNLGNNVNGNHLIPPAPPLPSQTDGAARGGTGHLISSTGALGSRSLF<br>SPLRNSMADSVDSRDIPGLPTNPSRLAAATSETCLLGGFEVLHDKGPLDILNT<br>QIGPSAFRVEVQADGTHAAIGEKNGLEVSVTLSPQEWSSLQSIDTEGKNRFV<br>FTGGRGGSGHPMVTVASDIAEARTKILAKLDPDNHGGRQPKDVDTRSVGVG<br>SASGIDDGVVSETHTSTTNSSVRSDPK**233**-*WSHPQFEK* |
| **NS-Tir** | *G***61**VDSRDIPGLPTNPSRLAAATSETCLLGGFEVLHDKGPLDILNTQIGPSAFR<br>VEVQADGTHAAIGEKNGLEVSVTLSPQEWSSLQSIDTEGKNRFVFTGGRGG<br>SGHPMVTVASDIAEARTKILAKLDPDNHGGRQPKDVDT**200**-*WSHPQFEK* |
| **SBB-Tir** | *G***76**LAAATSETCLLGGFEVLHDKGPLDILNTQIGPSAFRVEVQADGTHAAIGE<br>KNGLEVSVTLSPQEWSSLQSIDTEGKNRFVFTGGRGGSGHPMVTVASDIAE<br>ARTK**180**-*WSHPQFEK* |
| **C-Tir** | *GP***388**RRNQPAEQTTTTTTHTVVQQQTGGNTPAQGGTDATRAEDASLNRRD<br>SQGSVASTHWSDSSSEVVNPYAEVGGARNSLSAHQPEEHIYDEVAADPGYS<br>VIQNFSGSGPVTGRLIGTPGQGIQSTYALLANSGGLRLGMGGLTSGGESAVS<br>SVNAAPTPGPVRFV**550-***WSHPQFEK* |
| Human protein | |
| **SHP-1 C-SH2** | *GP***101**SDPTSERWYHGHMSGGQAETLLQAKGEPWTFLVRESLSQPGDFVLS<br>VLSDQPKAGPGSPLRVTHIKVMCEGGRYTVGGLETFDSLTDLVEHFKKTGIE<br>EASGAFVYLRQPYYATR**217**-*WSHPQFEK* |

[a]The first and last residues are in bold and extra residues due to cloning are in italics.

**Supplementary Table 5.** SAXS data collection and analysis.

| | N-Tir | NS-Tir | SBB-Tir | C-Tir |
|---|---|---|---|---|
| **Acquisition** | | | | |
| Beamline – Facility | B21-DSL | B21-DSL | B21-DSL | BM29-ESRF |
| Wavelength (Å) | 0.947 | 0.946 | | 0.992 |
| Sample-to-detector distance (m) | 2.694 | 2.696 | | 2.867 |
| *s* range ($Å^{-1}$) | 0.00032-0.37589 | 0.00034-0.43964 | | 0.00359-0.48896 |
| Concentration ($mg \cdot mL^{-1}$) | 20.0 | 25.0 | | 10.0 |
| HPLC system / SEC column | Agilent 1200 HPLC System / Shodex KW403-4F | Agilent 1200 HPLC System / Shodex KW402.5-4F | Agilent 1200 HPLC System / Shodex KW402.5-4F | Shimadzu HPLC System/ Superdex 200 Increase 3.2/300 |
| Detector | Pilatus 2M | Pilatus 2M | Pilatus 2M | Pilatus 1M |
| Temperature (K) | 298.15 | 298.15 | 298.15 | 298.15 |
| **Overall parameters** | | | | |
| $R_g$ (Å) [from *P(r)*] | 37.6±0.5 | 35.5±0.5 | | 38.7±0.4 |
| $R_g$ (Å) [from Guinier] | 37.7±0.5 | 35.5±0.5 | 17.8±0.8 | 38.8 ±0.4 |
| $D_{max}$ (Å) | 140±10 | 130±10 | 70±10 | 128±5 |
| Oligmeric state | 2-mer | 2-mer | 1-mer | 1-mer |
| **Software** | | | | |
| SEC-SAXS data integration | ScÅtter3 | ScÅtter3 | ScÅtter3 | ScÅtter3 |
| *P(r)* | GNOM 5.0 | GNOM 5.0 | GNOM 5.0 | GNOM 5.0 |
| *Ab initio* Modelling | NA[a] | DAMMIF | DAMMIF | NA[a] |
| **SASBDB accession code** | SASDKF8 | SASDKG8 | SASDRQ5 | SASDKH8 |

[a]Not applicable. Disordered/Flexible proteins are more accurately described as ensembles than single representations.

**Supplementary Table 6.** Primers used in this study for site-directed mutagenesis of C-Tir and full-length Tir.

| **Primer name** | **Primer sequence (5'→ 3')** |
|---|---|
| C-Tir_454F_Fwd | GAAGTAGTAAATCCCtttGCGGAAGTAGGGGGTGCG |
| C-Tir_454F_Rvs | CGCACCCCCTACTTCCGCaaaGGGATTTACTACTTC |
| C-Tir_474F_Fwd | CAGCCCGAAGAACACATCttcGATGAAGTAGCGGCC |
| C-Tir_474F_Rvs | GGCCGCTACTTCATCgaaGATGTGTTCTTCGGGCTG |
| Tir-_388E389E_Fwd | TGCGCTCCATgaagaaAATCAGCCGGC |
| Tir-_388E389E_Rvs | GTCGTTACACCAGCACCA |
| Tir-_513E_Fwd | TACTTATGCGgagCTGGCAAACAGCGG |
| Tir-_513E_Rvs | CTTTGGATACCTTGCCCTG |
| Tir_541*_Fwd | TGTAAATGCCtaaCCAACGCCGG |
| Tir_541*_Rvs | GAACTTACTGCGCTCTCG |
| Tir_CT_seq | CGATATGAGGATCAGCATGCC |

**Supplementary Table 7.** Plasmids used in this study

| **pICC number** | **Plasmid name** | **Plasmid description** |
|---|---|---|
| pICC1134 | pSA10-$Tir_{WT}$ | pSA10 plasmid encoding wild-type Tir from EPEC O127:H6 E2348/69 |
| pICC2931 | pSA10-$Tir_{388E389E}$ | pSA10 plasmid encoding EPEC Tir with R388E and R389E mutations |
| pICC2932 | pSA10-$Tir_{513E}$ | pSA10 plasmid encoding EPEC Tir with L513E mutation |
| pICC2934 | pSA10-$Tir_{541*}$ | pSA10 plasmid encoding EPEC Tir with a STOP codon replacing A541 |
| pICC2936 | pSA10-$Tir_{388E389E513E}$ | pSA10 plasmid encoding EPEC Tir with R388E, R389E and L513E mutations |
| pICC2938 | pSA10-$Tir_{388E389E513E541*}$ | pSA10 plasmid encoding EPEC Tir with R388E, R389E and L513E mutations and a STOP codon replacing A541 |

## Supplementary References


1. Fu, L., Niu, B., Zhu, Z., Wu, S. & Li, W. CD-HIT: accelerated for clustering the next-generation sequencing data. *Bioinformatics* **28**, 3150–3152 (2012).
2. Sievers, F. *et al.* Fast, scalable generation of high-quality protein multiple sequence alignments using Clustal Omega. *Mol. Syst. Biol.* **7**, 539 (2011).
3. Procter, J. B. *et al.* Alignment of Biological Sequences with Jalview. *Methods Mol. Biol.* **2231**, 203–224 (2021).
4. Elazar, A., Weinstein, J. J., Prilusky, J. & Fleishman, S. J. Interplay between hydrophobicity and the positive-inside rule in determining membrane-protein topology. *Proc Natl Acad Sci USA* **113**, 10340–10345 (2016).
5. Valentini, E., Kikhney, A. G., Previtali, G., Jeffries, C. M. & Svergun, D. I. SASBDB, a repository for biological small-angle scattering data. *Nucleic Acids Res.* **43**, D357-63 (2015).
6. Dosztányi, Z., Csizmok, V., Tompa, P. & Simon, I. IUPred: web server for the prediction of intrinsically unstructured regions of proteins based on estimated energy content. *Bioinformatics* **21**, 3433–3434 (2005).
7. Ishida, T. & Kinoshita, K. PrDOS: prediction of disordered protein regions from amino acid sequence. *Nucleic Acids Res.* **35**, W460-4 (2007).
8. Xue, B., Dunbrack, R. L., Williams, R. W., Dunker, A. K. & Uversky, V. N. PONDR-FIT: a meta-predictor of intrinsically disordered amino acids. *Biochim. Biophys. Acta* **1804**, 996–1010 (2010).
9. Jones, D. T. & Cozzetto, D. DISOPRED3: precise disordered region predictions with annotated protein-binding activity. *Bioinformatics* **31**, 857–863 (2015).
10. Giansanti, A., Deiana, A., Forcelloni, S. & Porrello, A. New classification of intrinsic disorder in the human proteome. *BioRxiv* (2018) doi:10.1101/446351.
