# Supplementary material for: The pathogen-encoded signalling receptor Tir exploits host-like intrinsic disorder for infection
Source: Commun Biol. 2024 Feb 13;7:179. doi: 10.1038/s42003-024-05856-9 (PMC10864410; doi:10.1038/s42003-024-05856-9)
Supplement: Supplementary file 2 — Description of Supplementary Materials [file 42003_2024_5856_MOESM2_ESM.docx]

**Description of Additional Supplementary Files**

**File name:** Supplementary Data 1

**Description:** Numerical source data for graphs in Figures 2 to 9
