# Supplementary material for: The pathogen-encoded signalling receptor Tir exploits host-like intrinsic disorder for infection
Source: Commun Biol. 2024 Feb 13;7:179. doi: 10.1038/s42003-024-05856-9 (PMC10864410; doi:10.1038/s42003-024-05856-9)
Supplement: Supplementary file 4 — Reporting Summary [file 42003_2024_5856_MOESM4_ESM.pdf]

nature portfolio

Corresponding author(s): Tiago N Cordeiro, COMMSBIO-22-3874

Last updated by author(s): Jan 6, 2024

Reporting Summary

Nature Portfolio wishes to improve the reproducibility of the work that we publish. This form provides structure for consistency and transparency in reporting. For further information on Nature Portfolio policies, see our Editorial Policies and the Editorial Policy Checklist.

Statistics

For all statistical analyses, confirm that the following items are present in the figure legend, table legend, main text, or Methods section.

n/a	Confirmed	
☐	☒	The exact sample size (*n*) for each experimental group/condition, given as a discrete number and unit of measurement
☐	☒	A statement on whether measurements were taken from distinct samples or whether the same sample was measured repeatedly
☐	☒	The statistical test(s) used AND whether they are one- or two-sided<br>*Only common tests should be described solely by name; describe more complex techniques in the Methods section.*
☒	☐	A description of all covariates tested
☐	☒	A description of any assumptions or corrections, such as tests of normality and adjustment for multiple comparisons
☐	☒	A full description of the statistical parameters including central tendency (e.g. means) or other basic estimates (e.g. regression coefficient) AND variation (e.g. standard deviation) or associated estimates of uncertainty (e.g. confidence intervals)
☐	☒	For null hypothesis testing, the test statistic (e.g. *F*, *t*, *r*) with confidence intervals, effect sizes, degrees of freedom and *P* value noted<br>*Give P values as exact values whenever suitable.*
☒	☐	For Bayesian analysis, information on the choice of priors and Markov chain Monte Carlo settings
☒	☐	For hierarchical and complex designs, identification of the appropriate level for tests and full reporting of outcomes
☒	☐	Estimates of effect sizes (e.g. Cohen's *d*, Pearson's *r*), indicating how they were calculated

Our web collection on statistics for biologists contains articles on many of the points above.

Software and code

Policy information about availability of computer code

Data collection	DISOPRED 3.0 and IUpred 1.0 were used to compute disorder propensities;<br>SEC data was collected using UNICORN;<br>We applied TopSpin (Bruker) to acquire NMR data;<br>We run MD simulations with GROMACS;<br>We used AlphaFold2.0 to predict the structure of the NS-Tir folded region using default settings implemented in LocalColabFold;<br>We used AlphaFold2-multimer to predict the C-Tir:C-SH2 complex;<br>We used Flexible-Meccano and SCCOMP to generate realistic structural ensembles of disordered regions;<br>We used CRYSOL to simulated the SAXS pattern from PDB files;<br>Immunofluorescence microscopy data acquisition was collected using a Zeiss Axio Imager Z1 microscope (Carl Zeiss).
Data analysis	NMRPipe, TopSpin and CARA were used in NMR data processing and analysis;<br>We used nsSPC, CheSPI and N-Talos to provide structural information from chemical shiffts.<br>Relaxation rates were analyzed with CcpNmr AnalysisAssign.<br>We exploit the state-of-the-art capabilities of ScÅtter and the ATSAS software suite to analyze and process SAXS data;<br>Structural ensembles were refined against PRE/SAXS data with EOM;<br>Plots were generated with Python, Seaborn, Veusz and and GraphPad Prism 5.1.<br>Molecular graphics and analyses were performed in ChimeraX-1.4;<br>Multiple sequence alignments were performed in Clustal Omega. Alignments were generated in Jalview.

For manuscripts utilizing custom algorithms or software that are central to the research but not yet described in published literature, software must be made available to editors and reviewers. We strongly encourage code deposition in a community repository (e.g. GitHub). See the Nature Portfolio guidelines for submitting code & software for further information.

Data

Policy information about availability of data

All manuscripts must include a data availability statement. This statement should provide the following information, where applicable:

- Accession codes, unique identifiers, or web links for publicly available datasets
- A description of any restrictions on data availability
- For clinical datasets or third party data, please ensure that the statement adheres to our policy

The NMR chemical shifts of C-Tir, pC-Tir and NS-Tir are available at the BMRB with accession codes 50758 (C-Tir), 50759 (pC-Tir) and 52057 (NS-Tir). The SEC-SAXS data and models are available at SASBDB) 122 under the project "SAXS studies on the intracellular region of the translocated intimin receptor". The accession codes are detailed in Supplementary Table 5. Proteomes and effector collections, disorder predictions are available for download at https://osf.io/3mka9/ and supplementary material. The associated code is available at https://osf.io/cxkjf/. A PRE/SAXS-based structural sub-ensemble of 200 structures for C-Tir is in the open-access Protein Ensemble Database (PED) 123 with the identifier code PED00210.

Human research participants

Policy information about studies involving human research participants and Sex and Gender in Research.

Reporting on sex and gender	NA
Population characteristics	NA
Recruitment	NA
Ethics oversight	NA

Note that full information on the approval of the study protocol must also be provided in the manuscript.

Field-specific reporting

Please select the one below that is the best fit for your research. If you are not sure, read the appropriate sections before making your selection.

☒ Life sciences ☐ Behavioural & social sciences ☐ Ecological, evolutionary & environmental sciences

For a reference copy of the document with all sections, see nature.com/documents/nr-reporting-summary-flat.pdf

Life sciences study design

All studies must disclose on these points even when the disclosure is negative.

Sample size	No statistical methods were used to determine sample size. All assays were performed in biological triplicate as is standard in the field and the data obtained had passed normality tests prior to further statistical analyses.
Data exclusions	Data were not excluded from analysis.
Replication	SAXS experiments were performed in duplicates at ESRF-BM29 and DLS-B21 Bio-SAXS beamlines.<br>NMR experiments were performed using technical replicates. For practical reasons, biological replicates are not standard practice in the field.<br>Cell-based assays were performed with at least 3 biological replicates.
Randomization	Randomization was not performed as it is not standard practice in the field.
Blinding	Blinding is not relevant to this study as all results could be obtained objectively. No subjective measurements were recorded in this study.

Reporting for specific materials, systems and methods

We require information from authors about some types of materials, experimental systems and methods used in many studies. Here, indicate whether each material, system or method listed is relevant to your study. If you are not sure if a list item applies to your research, read the appropriate section before selecting a response.

Materials & experimental systems

n/a	Involved in the study
☐	☒ Antibodies
☐	☒ Eukaryotic cell lines
☒	☐ Palaeontology and archaeology
☒	☐ Animals and other organisms
☒	☐ Clinical data
☒	☐ Dual use research of concern

Methods

n/a	Involved in the study
☒	☐ ChIP-seq
☒	☐ Flow cytometry
☒	☐ MRI-based neuroimaging

Antibodies

Antibodies used	Donkey polyclonal anti-Rat IgG (H+L) conjugated to Alexa Fluor 488: Jackson Immunoresearch Cat# 712-546-150 used at 1:200. Rabbit anti-EPEC O127:H6 serum (VLA) used at 1:1,000.
Validation	Based on immunoelectrophoresis and/or ELISA, the antibody reacts with whole molecule rat IgG. It also reacts with the light chains of other rat immunoglobulins. No antibody was detected against non-immunoglobulin serum proteins. The antibody has been tested by ELISA and/or solid-phase adsorbed to ensure minimal cross-reaction with bovine, chicken, goat, guinea pig, syrian hamster, horse, human, rabbit and sheep serum proteins, but it may cross-react with immunoglobulins from other species.

Eukaryotic cell lines

Policy information about cell lines and Sex and Gender in Research

Cell line source(s)	SNU-C5 cells
Authentication	None of the cell lines have been authenticated.
Mycoplasma contamination	Cell line was tested free of Mycoplasma contamination
Commonly misidentified lines (See ICLAC register)	No commonly misidentified cell lines were used.
